# Expanding school counseling: The impacts of California funding changes

**DOI:** 10.1016/j.econedurev.2025.102754

**Published:** 2026-01-03

**Authors:** Daniel Sparks, Christine Mulhern

**Affiliations:** a University of Arkansas, United States; b RAND, United States

**Keywords:** School Counseling, School Counselors, Educational Policy, Program Evaluation, High School Students, Counseling Services, Counselor Qualifications, Graduation Rate, College Attendance, Academic Advising, Student Attitudes, Educational Environment

## Abstract

Counselors are a common school resource for students navigating complicated and consequential education choices, but most students have limited access to them. We study one of the largest U.S. policies to increase access to school counselors. We use a variety of panel-based estimators to show that California’s Supplemental School Counseling Program increased the number of counselors on staff by about one and improved counselor to student ratios, but reduced average counselor experience. We find that increased funding for counselors led to large increases in high school graduation and measures of school climate. The policy also led to more modest gains in California high school exit exam performance and some public college enrollment rates. These impacts were largest at high poverty and rural schools, as well as for students who were male, socioeconomically disadvantaged, or Black or Hispanic. Thus, expanding access to counselors may help address equity gaps in college access and student well-being.

## 1. Introduction

School counselors can be important resources for students, helping them navigate educational options, develop social and emotional skills, and succeed in and beyond school (Carrell & Hoekstra, 2014; Mulhern, 2023; Reback, 2010). Despite their potentially important role, most students have limited access to counselors. On average, public school counselors serve over 400 students per year and typically spend only a few hours with each student. Recently, there has been a push at federal and state levels to increase funding for and access to school counselors. For instance, many states used some of their federal COVID-19 relief funding to hire more counselors, and California is pushing to double its number of school counselors (Modan, 2022; Prothero & Riser-Kositsky, 2022). However, little research examines how to effectively design policies for expanding access to school counseling and the impacts of expanded access to school counselors on students.

We study one of the largest policies to increase funding for school counselors. Starting in 2006, California’s Supplemental School Counseling Program provided block grants totaling $200 million to schools to support increased access to middle and high school counselors. The legislation required the funds “supplement, and not supplant expenditures” for school counseling programs, and “provide supplemental counseling services delivered by personnel who hold a valid pupil personnel services credential” (Supplemental School Counseling Program, 2007). Over 1000 additional counselors were hired in the first two years of the program, and student-to-counselor ratios fell precipitously. In 2009, the spending guidelines were eliminated and schools no longer had to spend funds specifically on counselors. The investments in school counseling could improve student outcomes by increasing access to counselors, however, the rapid hiring of counselors may have had negative effects if it pulled less experienced counselors into the profession (Jepsen & Rivkin, 2009; Mulhern, 2023).

We study the funding, equity, and achievement implications of the funding expansion. We use student, school, and educator data from the California Department of Education to study how schools responded to the policy and funding changes, and the subsequent impacts on student outcomes. Overall, we find that schools used the funding to hire more school counselors, with the average high school hiring about one additional counselor with the funding. This represented an average increase in the number of counselors per 1000 students of .578 (or a decline in the student-to-counselor ratio of roughly 150 students per counselor). Schools in non-rural areas and those serving a larger student body were most likely to hire counselors. We also find that the program funding led to a reduction in the average counselor’s experience by about three years largely by leading to the hiring of less experienced counselors.

We use an event study design to examine how access to school counselors and student outcomes changed when the policy was implemented. We leverage variation over time and across schools to compare outcomes for cohorts of students that were and were not exposed to the increase in counselor funding. We find that increased funding for counselors led to a 3 percentage point increase in high school graduation rates, with more modest effects on high school exam pass rates of less than a percentage point. There is also some indication that the program may have increased enrollment rates at community colleges and University of California (UC) schools and led to declines at California State University (CSU) schools suggesting that counselors may shift marginal and inframarginal students to attend different types of institutions. Furthermore, we find that increased funding for school counselors led to substantial improvements in students’ perceptions of school climate, which could impact academic achievement (Berkowitz, Moore, Astor, & Benbenishty, 2017).

The program had larger effects on students who were male, Black or Hispanic, or socioeconomically disadvantaged, as well as in high poverty and rural schools. Pass rates on the high school exit exams increased by about 3 percentage points for students who were Black or Hispanic or from a socioeconomically disadvantaged background. In addition, UC enrollment increased by a percentage point in high poverty schools, and overall public college enrollment increased by 5 percentage points in rural schools. At low poverty schools, expanding access to counseling appears to have shifted some students towards attending more selective public colleges. These results are consistent with research on high school counselors in other settings and with the general goals of the program (Mulhern, 2023; Supplemental School Counseling Program, 2007). This is one of the first studies to examine causal effects of a large-scale investment in school counselors on student outcomes, and offers insights into the efficacy of statewide policies geared toward improving access to school counselors.

The paper proceeds as follows. Section 2 discusses the policy background and relevant literature. Section 3 presents the data, and Section 4 describes the empirical approach. Section 5 presents descriptive and main results, Section 6 includes a discussion, and Section 7 concludes.

## 2. Background & literature review

### 2.1. Policy background

A 2006 report on California Educational Opportunity called out the state’s declining performance in terms of high school graduation and college access relative to other states, noting limited access to rigorous curriculum, disparities in access to quality teachers, and a lack of adequate (or any) college advising as contributing factors (Rogers, Terriquez, Valladares, & Oakes, 2006). In the early 2000s, less than a third of California’s high school graduates matriculated to a four-year college. Policymakers were concerned about declines in the percentage of students enrolling in California State Universities (CSU) and University of California (UC) schools. Thus, policymakers turned their attention to school counselors as a potential remedy for improving college access and reducing persistent racial equity gaps (Rowell, Whitson, & Thomas, 2008).

California State Assembly Bill 1802 established the Supplemental School Counseling Program (SSCP) in 2006 to make available additional state funds for districts to spend on school counselors. Funds were allocated based on reported enrollment and had to be used specifically for school counseling programs. Funds from the SSCP could not supplant extant school district expenditures on counselors or be used on non-counseling inputs (Supplemental School Counseling Program, 2007). Districts that opted to receive program funds were required to ensure that all students had access to a school counselor and received individualized reviews of academic and career goals (Supplemental School Counseling Program, 2007). Other notable provisions of the program include the requirement for districts receiving funds to develop a high school transition and college preparation curriculum and ensure that students meet with a school counselor at least once during grades 7, 10 and 12 (Supplemental School Counseling Program, 2007). The program stipulated that counseling services be prioritized for students at risk of failing out of school or who had already failed the California State High School Exit Exam (CAHSEE).

As a result, we expect to find reductions in achievement gaps and improvements in the percentage of students scoring proficient on the exit exam. The high school class of 2006 was the first cohort required to pass the exit exam in order to graduate. These students would have first taken the exam as 10th graders in spring 2004. Thus, three cohorts of 10th graders took the exam before the SSCP was implemented. If a student failed one or multiple sections of the exam during their sophomore year, they were allowed to retake failed sections of the exam in subsequent years. Per language in SSCP legislation, school counselors were encouraged to help at-risk students enroll in or retake exit exams and may also have informed or reminded students of the requirement to pass exit exams in order to graduate from high school.

The policy contained limited restrictions for school district receipt of funds or accountability, and more than 90 percent of districts received SSCP funding prior to program termination. Funding allocations were based on student enrollment, with the total annual SSCP funding of $200 million working out to $79 in additional per student spending for counseling services. While the money had to be used on counseling services, schools took a number of approaches to spending SSCP funds. For instance, some districts used funds to hire new counselors while others used the money to increase counseling services specifically for students at-risk of dropping out. With regard to accountability, the California Department of Education asked school districts to provide an annual report including data on relevant student outcomes and metrics on student-to-counselor interactions, but receipt of funds or funding amount was not dependent on year-over-year results presented in these reports (Zubko, 2010).

Both the policy stakeholders who advocated for the law and the law itself indicate that the SSCP targeted lowering student-to-counselor ratios as a means of improving high school graduation rates, college preparation, and matriculation to California colleges and universities, decreasing persistent equity gaps in college access, and improving school climate. This study examines whether the legislation achieved its intended goals and how the school counseling workforce changed during this time period.

### 2.2. Literature review

There is extensive literature on the importance of school spending on student outcomes (e.g., Jackson, Johnson, & Persico, 2016; Lafortune, Rothstein, & Schanzenbach, 2018), but relatively little is known about the effects of investments in particular school resources. Extant research that has looked into effects of large-scale investments in specific school inputs tends to focus on classroom teachers and class size (e.g. Chetty, Friedman, & Rockoff, 2014; Fredriksson, Öckert, &Oosterbeek, 2013; Krueger, 1999), whereas very little is known about the effects of similar, large-scale investments in school counselors.

The SSCP increased funding for school counselors to improve students’ likelihood of interacting with a counselor as well as the quality of interactions. Understanding the impact of investments in school counseling is particularly important given the recent evidence on the impacts of school counselors on educational attainment (Mulhern, 2023). There is some evidence that expanding access to elementary and middle school counselors improves behavior and test scores for subgroups of students (Carrell & Hoekstra, 2014; Reback, 2010). However, these papers focus on small policy changes for counselors with very large caseloads. One of them focuses on hiring counseling interns to lower caseloads and neither study high school students, for whom we may expect counselors to be especially important. For instance, we expect reduced student-to-counselor ratios in California high schools to improve the proportion of students passing high school exit exams and graduating.

Moreover, successfully navigating the college application process requires a tremendous amount of information, preparation, and advising to complete, for instance, FAFSA applications, requisite standardized tests, essays and college applications (Dynarski, Page, & Scott-Clayton, 2022). Complexity in the college application process may disproportionately act as a barrier to college access for low-income students and students of color in the U.S. despite these students reporting similar expectations around college-going to their white and higher-income peers (Perna & Titus, 2005). Similarly, information constraints are more likely to bind for students who lack access to social and cultural capital; counselors may help to relax these constraints by supporting students as they navigate their academic and professional plans post-high school (Dynarski, Libassi, Michelmore, & Owen, 2021; Dynarski et al., 2022; Hoxby & Turner, 2013; Mulhern, 2021).

Some prior research has found that counselors influence whether and where students attend college (Hurwitz & Howell, 2014; Mulhern, 2023). Thus, we anticipate that the SSCP may have increased college enrollment and potentially shifted the types of institutions that students attended. We further anticipate improved counselor-to-student ratios to boost the percentage of students completing college preparatory exams such as the SAT since counselors can help to notify students of these exams, help register them, and provide fee waivers. Expanded access to counseling may also improve student test scores if expanding counseling services helps counselors better assign students to appropriate courses, provide accommodations, or support student needs that can impact learning.

While prior work examines the importance of high school counselor caseloads, estimates from these papers are very noisy and subject to important limitations (Hurwitz & Howell, 2014; Mulhern, 2023). Hurwitz and Howell (2014) leverage minimum counseling ratios in 12 states in a regression discontinuity design and find large (but noisy) effects on four-year college enrollment, though they are unable to measure impacts on other student outcomes. Mulhern (2023) looks at smaller changes in caseloads in Massachusetts using year to year variation in the number of counselors and students within schools. She finds limited changes in student outcomes associated with changes in counseling ratios, but it is unclear if these estimates are causal and if larger changes may be associated with larger changes in access to school counselors. There are also several papers documenting a negative correlation between counselor caseloads and academic achievement (e.g., Gagnon & Mattingly, 2016; Lapan, Whitcomb, & Aleman, 2012; Woods & Domina, 2014). Still, it is unclear if these estimates simply pick up on the fact that higher resourced schools, with students from higher income backgrounds, tend to have counselors with smaller caseloads.

School counselors may also be an important resource for improving school climate. While the empirical literature evaluating counselor effects on school climate is thin, prior studies highlight the positive relationship between school climate and students’ academic outcomes (e.g., Berkowitz et al., 2017; Thapa, Cohen, Guffey, & Higgins-D’Alessandro, 2013), though it again remains unclear if this relationship is causal (Benbenishty, Astor, Roziner, & Wrabel, 2016; Kraft, Marinell, & Shen-Wei Yee, 2016). We build on this work by providing some of the first causal evidence of how expanding spending on and access to high school counselors impacts perceptions of school climate. Counselor responsibilities vary and extend well beyond activities related to college preparation; meeting with students experiencing social and emotional issues is often a core part of counselors’ job descriptions (NACAC, 2025). Greater counselor availability could improve the number and quality of interactions with students, which could help counselors provide more targeted or individualized interventions. Both of these counselor effects should improve mental health resources available for students and school climate outcomes, which may contribute to increases in observable academic outcomes.

Finally, this work builds on studies such as Jepsen and Rivkin (2009) which study the general equilibrium effects of large scale public policies. Since we study a statewide policy change, rather than an increase in the number of counselors in a few schools, our estimates account for how such a large policy may change the types of counselors to which students have access. Importantly, the SSCP maintained counselor licensure standards, so the quality of counselors in terms of credentialing should remain unchanged. However, the policy could have led to the hiring of less experienced counselors since it was unclear whether there was sufficient supply of experienced counselors to meet the rapid growth in demand spurred by the policy. While funds received from SSCP had to be spent on counseling services, school districts had discretion in hiring decisions, which is likely to impact variation in effect sizes on outcomes of interest. Our findings shed light on policy effects on both the counselor labor market and student outcomes and hold timely recommendations for policymakers as schools look for ways to reduce equity gaps in college access and address student mental health concerns coming out of the COVID-19 pandemic.

## 3. Data & analytic sample

We construct a school-by-year level dataset using publicly available data from the California Department of Education (CDE). CDE data include school enrollment by grade, race and gender, staff assignments and demographics, school financial records, and high school outcomes. We also have student-level data on high school student exit exam scores and student demographics from 2003–04 to 2012–13 school years. From staff assignment and demographic data, we are able to identify the number of counselors at each school and further disaggregate this information by race/ethnicity, gender, and years of professional experience as a counselor both within the district and overall. School finance data include detailed records on school revenues and expenditures broken down by specific function and goals as reported through the Standardized Account Code Structure (SACS). This includes year-over-year school expenditures on, for instance, guidance and counseling services overall as well as expenditures made specifically through the Supplemental School Counseling Program.

Our main high school academic outcomes of interest are high school graduation rates and California High School Exit Exam (CAHSEE) pass rates and scores on the Math and English Language Arts (ELA) sections. The CAHSEE exam was standardized each year so the scores are comparable over time. We have restricted-access student level data on the CAHSEE exams while all other outcomes are measured at the school level. Policy effects of SSCP on high school graduation rates may not be apparent until a few years after the program started and the first cohorts of exposed 9th and 10th graders had time to graduate. Therefore, we focus on how high school graduation rates vary based on the year in which students were in tenth grade (the same year students take the CAHSEE).^1^ Unfortunately, the SAT changed in 2006, adding the writing section and substantially lengthening testing time. Since this change coincided with the timing of the SSCP, it may be difficult to separate the effects of the SSCP on SAT-taking from the effects of lengthening the SAT. We look at ACT-taking rates as well but encourage interpreting these results with caution.^2^

We also use a panel dataset of public high schools in California constructed by Lapid (2017) to capture the share of high school graduates who enroll in a California community college (CC), California State University (CSU), or University of California school (UC). These data were collected by the California Postsecondary Education Commission (CPEC) until the agency’s funding was disbanded in 2011. Lapid (2017) was able to construct the dataset up through 2009 using access to a data archive on the California Community College Chancellor’s Office website that is no longer maintained.^3^

To measure school climate, we use student-level data from the California Healthy Kids Survey. Many schools in California administer the survey biannually to 5th, 7th, 9th, and 11th grade students. We focus on responses for 9th and 11th graders at schools which administered the survey in at least one of our sample years prior to the SSCP (2003 to 2005) and one treatment year (2007 or 2008). We focus on responses to survey questions related to caring-staff relationships, school connectedness, and delinquency since these appear most related to school counselors’ roles. We construct indices of student responses based on the constructs and weights recommended and validated by Mahecha and Hanson (2020).^4^ We standardize each index so that all estimates are in terms of standard deviations. Table A.2 summarizes the survey questions included in each index.

**Fig. A.1.**
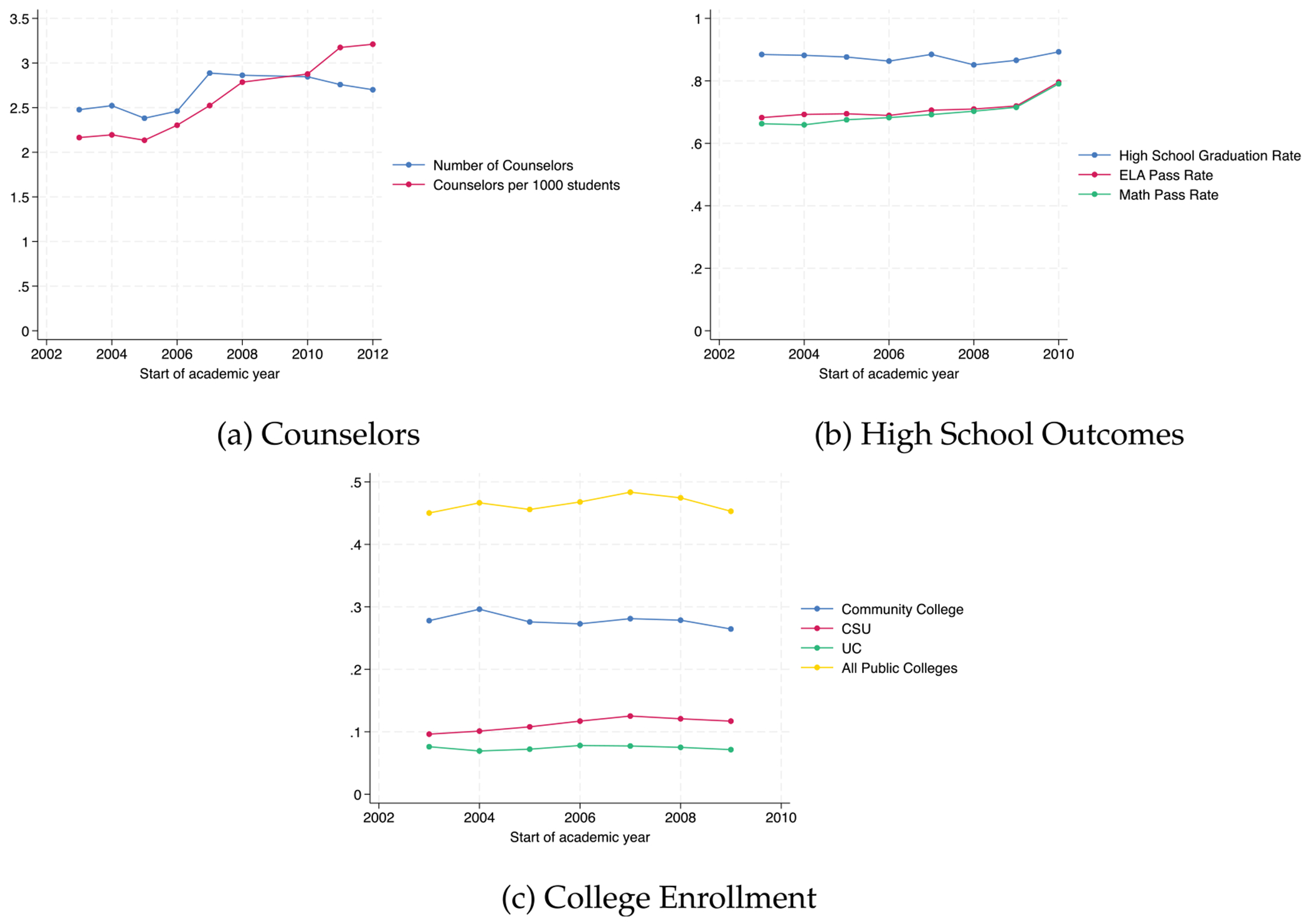
Trends in counselor & academic outcomes. Notes: Figures (a), (b), and (c) highlight trends in counselor and academic outcomes. College enrollment outcomes include community college enrollment, enrollment in a California State University institution, enrollment in a University of California institution, and overall public college enrollment.

Our main analytic sample focuses on students enrolled in California high schools from 2003–04 to 2012–13.^5^ Our focal measures of student outcomes are at the high school level, so we exclude middle schools from our main results. We also omit Los Angeles Unified School District from our analysis due to reporting errors described in Appendix B. Table 1 shows high school student demographics and relevant outcomes over time for more than 1000 high schools included in our sample; we present trends for the latter visually in the appendix (see Fig. A.1). This covers approximately 1.4 million students per year. Over our sample period average high school enrollment declined from 1328 in 2003 to 1196 in 2012. The share of Hispanic students increased by 11 percentage points over this same time period while the share of white students declined. The share of students receiving free or reduced-price lunch (FRPL) increased by 19 percentage points. High school graduation rates declined slightly after 2006, likely due to the new requirement for students to pass the CAHSEE exam to graduate high school. High school exit exam pass rates in ELA and math saw marginal gains while public college going was essentially flat.

Our student-level sample for analyses of the CAHSEE exam includes all 10th grade students in California public schools between 2003–04 and 2010–11 who have a valid CAHSEE exam record from 10th grade.^6^ This sample includes 2.6 million students, but we primarily focus on the 2.2 million students in tenth grade between 2003–04 and 2008–09.^7^ The same restrictions apply for which schools/districts are included as with the school level sample.^8^

Table A.3 summarizes the 416 schools and 1,037,473 students in our sample for the California Healthy Kids Survey. Since not all students and schools administered the survey, and because it is only administered in some grades, this sample is smaller and slightly different from our main sample. In particular, students and schools in this sample are more likely to be Asian, in urban areas, and to have higher achievement levels as measured by the CAHSEE exam scores and high school graduation rates than students in the main sample. We limit our sample to schools in which the survey was administered in at least one study year prior to the SSCP (2003–2005) and one year after initial SSCP implementation (2007–2008). Schools included in the survey sample vary slightly from year to year and response rates within schools may also vary over time. Table A.4 shows how characteristics of the survey respondents vary over our study period. To address some of this variation, we include school fixed effects and control for student demographics (age, race/ethnicity, and gender); we offer more expansive details on our identification strategy in the following section.

## 4. Empirical approach

Next, we describe our approach for estimating the causal effects of the program on academic and school climate outcomes. We leverage the sharp timing of the SSCP policy in an event study design which compares changes before and after the policy implementation in 2006 while controlling for statewide trends in student outcomes. We include school fixed effects to account for differences across high schools such as other services offered and community factors. To account for potential time trends leading up to the SSCP (e.g., increasing rates of Hispanic students) we fit a parametric event study specification as in Dobkin, Finkelstein, Kluender, and Notowidigdo (2018), Freyaldenhoven, Hansen, and Shapiro (2018), and Rambachan and Roth (2023). We chose this empirical specification because it fits our data better than alternatives, including a simple two-way fixed effects event study model or a model with school specific time trends.^9^ Freyaldenhoven et al. (2018), and Rambachan and Roth (2023) each indicate that controlling for pre-period trends in this way is a valid approach for estimating event study models.

To implement this approach, we identify a functional form that fits the data in the pre-period (above and beyond what is captured by the school fixed effects) and then control for the expected trend over the entire time period (Rambachan & Roth, 2023). As in Dobkin et al. (2018), we fit this in a two-step process. First, we fit the following regression for all observations between 2003 and 2005.

(1) $$Y_{st}={\alpha Time}_{t}+\gamma_{s}+\epsilon_{st}$$

Here, an outcome, *Y_st_*, is defined for students in school *s* in year *t*, *γ_s_* is school fixed effects and *Time_t_* is a linear variable for time from the policy implementation. We then compute the residuals (${\bar{Y}}_{st}$) as the difference between the predicted values (${\hat{Y}}_{st}$) and *αTime_t_*. The residuals are the estimated effects absent the time trend but inclusive of the fixed effects. This nets out the time trend (based on *Time_t_*) in the post-period that one would expect based on any trend observed in the pre-period. Then, we fit the following equations with our residuals (${\bar{Y}}_{st}$).

(2) $${\bar{Y}}_{st}={\beta Year}_{t}+\gamma_{s}+X_{st}+\epsilon_{st}$$

(3) $${\bar{Y}}_{st}=\delta SSCP_{st}+\gamma_{s}+X_{st}+\epsilon_{st}$$

In Eq. (2), *Year_t_* is a vector with indicators for each year, and *β* is a vector of coefficients indicating how the outcome varies for each time period. This equation is used to plot our event study estimates, with a separate estimate for each time period. We fit this model for all years from 2003 to 2012, except where data are not available for the full time period.^10^

We cluster standard errors at the school by year level; though results are similar when clustering at the school and district levels (see Table A.5). We weight school-level regressions by the number of students enrolled in grades 9 through 12. We include controls, *X_st_*, for school racial and gender composition, percent of students receiving free or reduced-price meals, urbanicity, and instructional spending in our figures and main models, and also show estimates without these controls.^11^ The student level models include controls for individual student race/ethnicity, gender, as well as school urbanicity, share of students receiving free or reduced-price lunch, and instructional spending. Estimates based on the student-level CAHSEE data also control for an indicator for socioeconomic disadvantage, and models based on the survey data include controls for student grade level and age.^12^ Because of some changes in survey respondents over time, we focus on the survey estimates that include controls for student (and school) characteristics.

Our main results allow for variation in implementation timing across schools. The majority of schools first received SSCP funding in 2006, but some schools first received funds in 2007 or 2008. Thus, our main models focus on time relative to when a school first received SSCP funds.

Eq. (3) is used to estimate the average effect of the policy. Here, *SSCP_st_* is an indicator which equals one in the years after SSCP implementation and *δ* is the average effect of the policy. We fit this model using three years of pre-period data (2003–2005) and two years of post-period data (2007–2008). We exclude 2006 from this model because the event study plots associated with Eq. (2) suggest it took a year for the policy to be implemented and have any effects. We also exclude 2009–2012 from the average effects because the policy differed in these years.

Our event study design will yield valid causal estimates so long as there were no anticipatory effects prior to the SSCP and outcomes would have evolved in a similar fashion after the SCCP was implemented if not for the program. It is unlikely that there were any anticipatory effects because there were only a few months between when the bill passed and the program began, and no SSCP funding was available to hire counselors until after policy implementation. There are some other widespread changes which could affect a few of our outcomes. For instance, the state changed high school graduation requirements so that, beginning with the class of 2006, all students had to pass the CAHSEE in order to graduate. This potentially influenced high school graduation rates and may have influenced passing rates on the CAHSEE. To address this, we focus on CAHSEE pass rates for 10th graders rather than overall pass rates since changes in graduation requirements likely had the largest effect on 11th and 12th graders retaking the exam to meet graduation requirements. We also focus on the high school graduation rates for cohorts of 10th graders — e.g., we treat the 2006 10th graders as the first treated cohort since the CAHSEE was likely the main mechanism through which counselors influenced graduation. In addition, the SAT added the writing component in 2006 which may have decreased SAT taking. To address this limitation we look at ACT taking as well since some students may have substituted the ACT for the SAT. However, without student level data it is hard to measure substitution between exams, so we only present these results in the appendix. Finally, the SSCP included guidance around the academic advising students should receive. Thus, our estimates reflect the impact of the entire SSCP program and may not necessarily indicate the independent effect of expanding access to school counselors without guidance about the counseling they are intended to provide.

This event study design controlling for trends in the pre-period is our preferred model in this policy context for two central reasons. First, a number of outcomes of interest exhibit linear trends in the pre-period, resulting in a failure of the unconditional parallel trends assumption. We present event study results from models with school by year fixed effects instead of controlling for linear trends in the pre-period in Fig. A.3; these results affirm increases in counselors and counselors per 1000 students after SSCP was implemented, but also highlight evidence of linear pre-trends for high school graduation and college-going outcomes. Second, the SSCP had high take up rates; while this is positive from a policy perspective in terms of documenting disbursed funding for counselors, it also means that we are left with a small number of districts and schools that can serve as a pure control group. We include several robustness checks to assess the validity of our identification strategy and findings. We use an instrumental variables approach, where per student SSCP spending is instrumented on student to counselor ratios (see Table A.6). To address potential concerns around staggered treatment adoption, we show robustness to an intent to treat approach where everyone’s treatment is defined to occur at the same time - i.e. starting in 2006 (see Tables A.7 and A.8). Additional appendix tables show our estimates including 2006 as a treated year (see Tables A.9 and A.10). To address concerns with two-way fixed effects models and model specifications that control for linear pre-trends, we include results from a semi-parametric two-way fixed effects approach that accounts for variation in treatment timing (Callaway & Sant’Anna, 2021; Rios-Avila, Sant’Anna, & Callaway, 2023) and includes high schools that were not yet treated or never received SSCP funds as a comparison group (see Table A.11). Last, we run heterogeneity analysis to better understand how estimated effects vary across school, student and counselor characteristics as well as by continuous measures of SSCP spending and for other school climate survey results (see Tables A.12, A.13, A.14, A.15, and A.16).

## 5. Results

### 5.1. Descriptive results

We first describe how schools responded to SSCP funding and whether the legislation had its intended effects on access to school counseling. In 2006, 88 percent of the districts in our sample received SSCP funds; this jumped to 95 percent of districts in 2007 and fell to 77 percent in 2008. Table 2 shows the difference in high school student demographics and outcomes for districts that used SSCP funds versus those that did not. Compared to the relatively few districts that did not use SSCP funds, districts that participated in the program were less likely to be located in rural areas, served a lower percentage of students eligible for free or reduced price lunch, and had higher SAT participation rates and public college going rates. Districts that used SSCP funds had a comparable ratio of counselors per 1000 students compared to districts that did not receive SSCP funds.

**Fig. 1.**
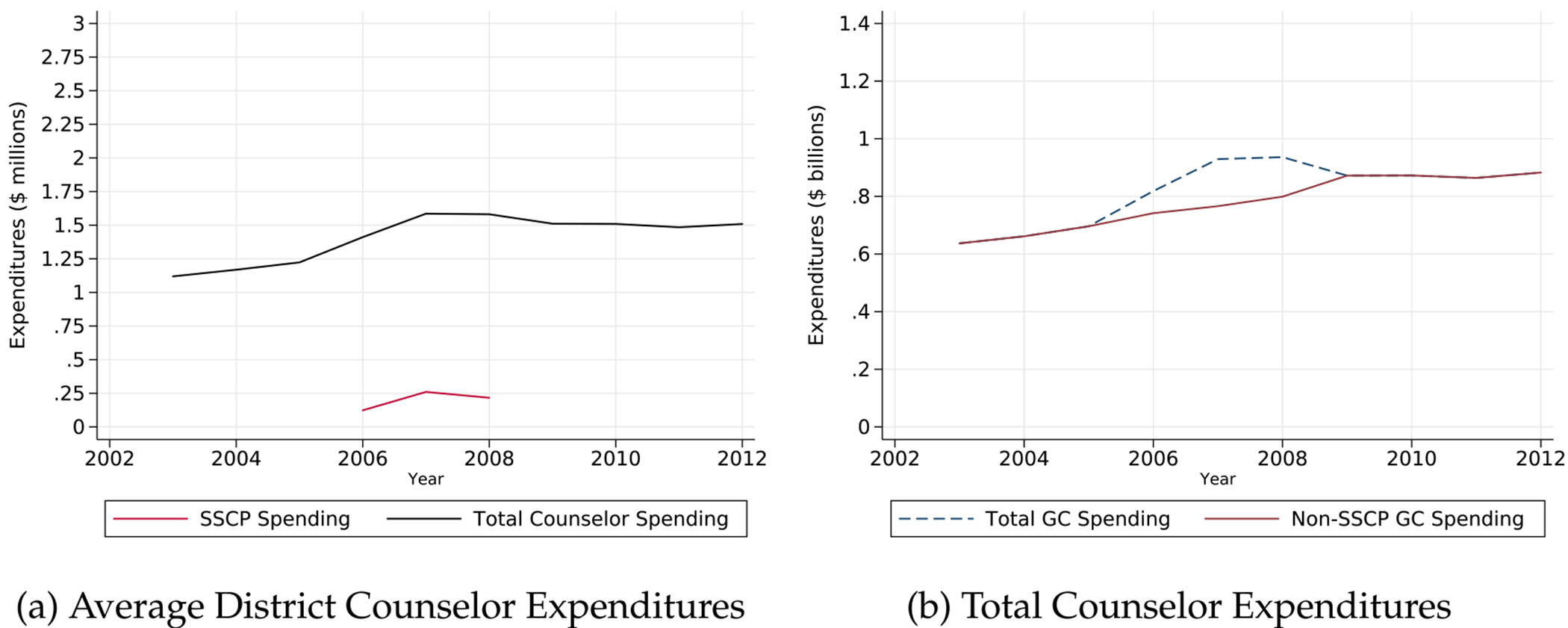
Trends in counselor spending. Notes: Figures display (a) average district counselor expenditures in California, including total counselor spending and spending made more specifically through SSCP funding and (b) total counselor expenditures across all districts in our analytic sample, inclusive and exclusive of SSCP funding from 2003–2012.

**Fig. A.2.**
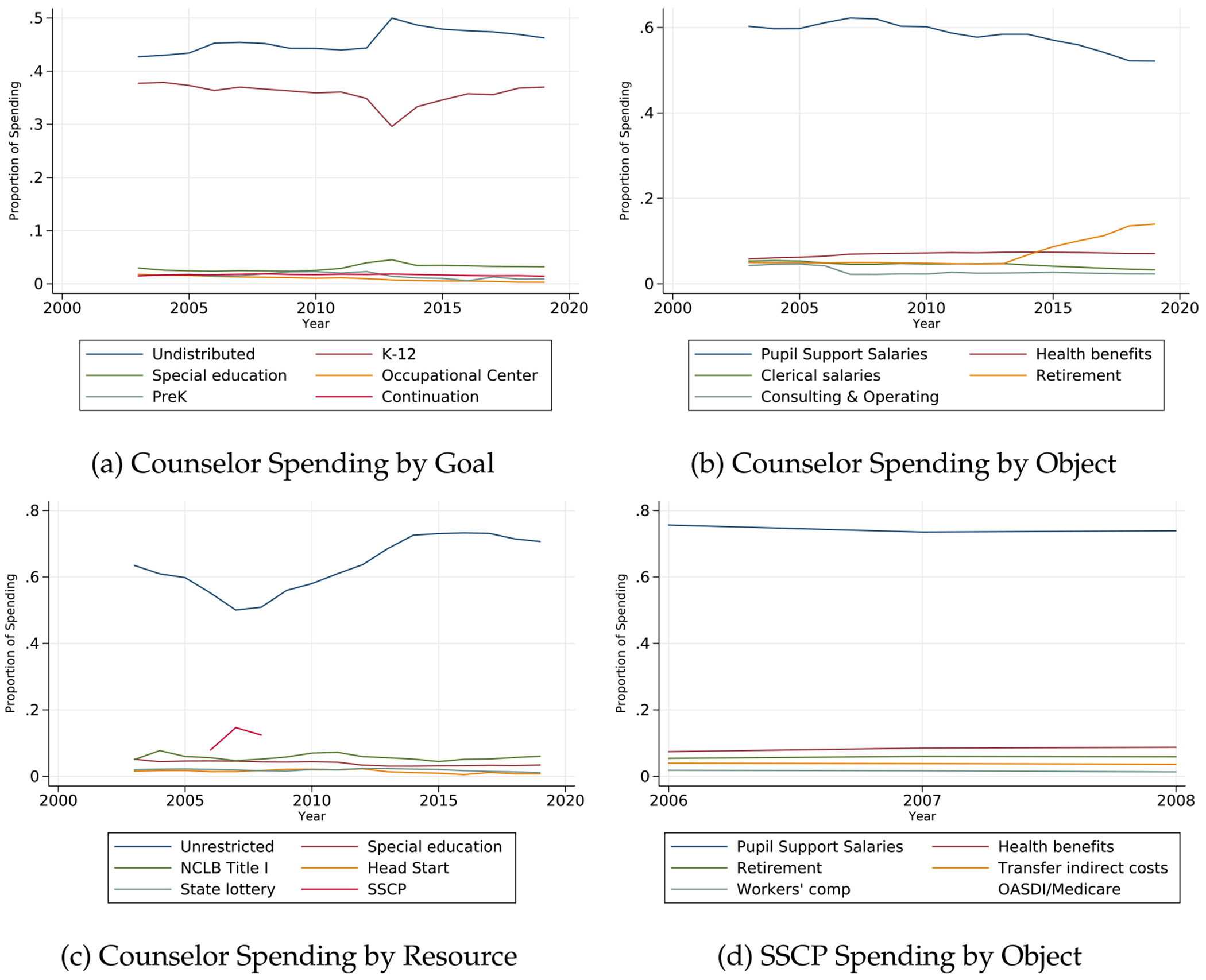
Trends in guidance counselor spending. Notes: Figures (a), (b), and (c) highlight trends in guidance counselor spending as broken down by spending goal, object, and resource as reported by California Department of Education (CDE). Figure (d) similarly shows SSCP spending broken down by spending object.

**Fig. A.3.**
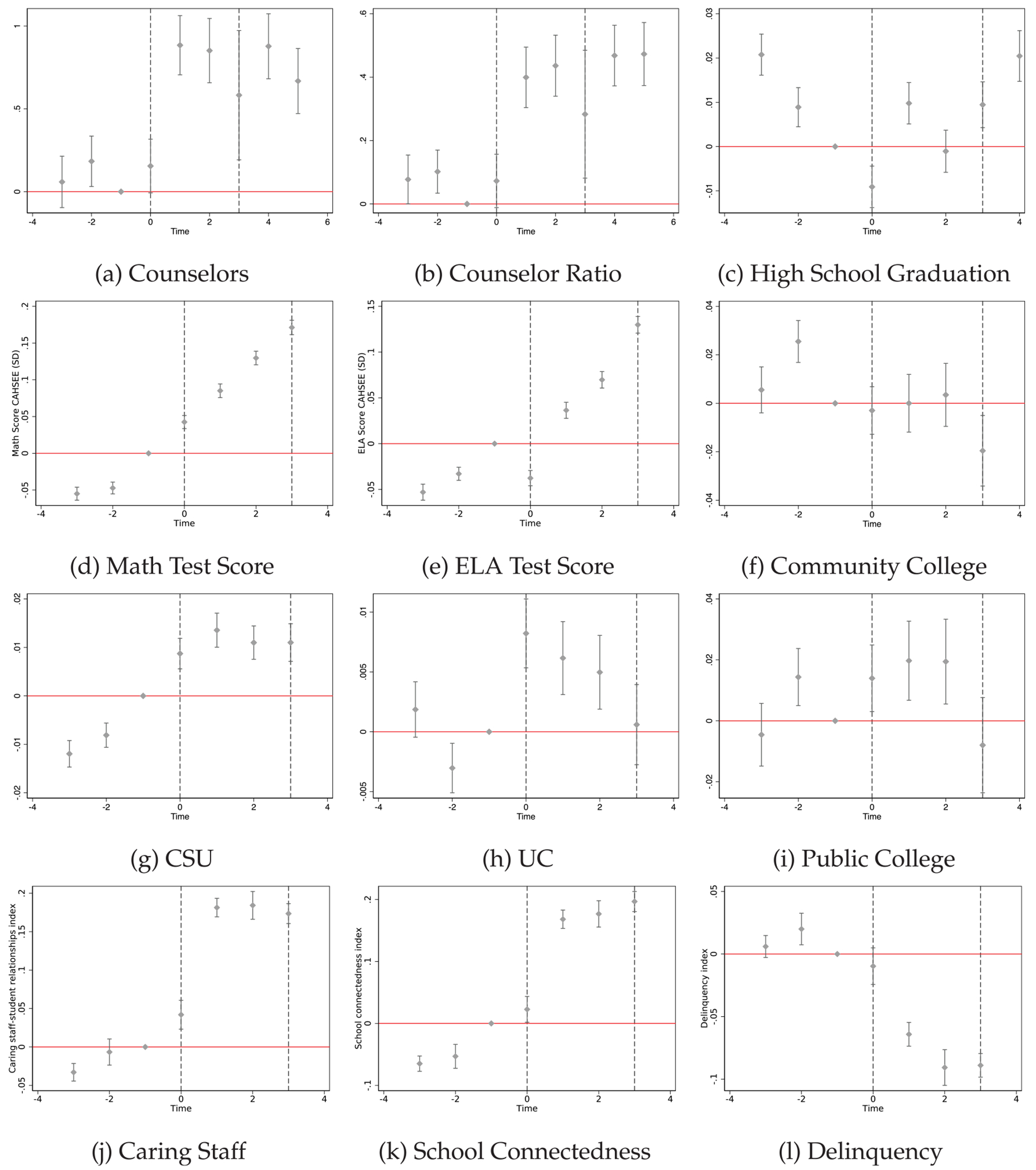
Event study results using school specific time trends. Notes: Event study results are depicted using models school by year fixed effects and controlling for school demographic characteristics and instructional spending. Outcomes depicted include the number of counselors on staff, counselors per 1000 students, high school graduation rate, and the share of students enrolling in a California community college, California State University, University of California institution, or any California public college.

In our sample, average district expenditures on guidance counseling increased by about a quarter of a million dollars as a result of SSCP. Fig. 1(a) shows that districts spent over $1.5 million on average during SSCP program years before declining in 2010. Similarly, Fig. 1(b) shows that total counseling expenditures increased by about $200 million before declining in 2010. The vast majority of counselor spending overall and through SSCP goes toward salaries and health benefits (see Fig. A.2).

**Fig. 2.**
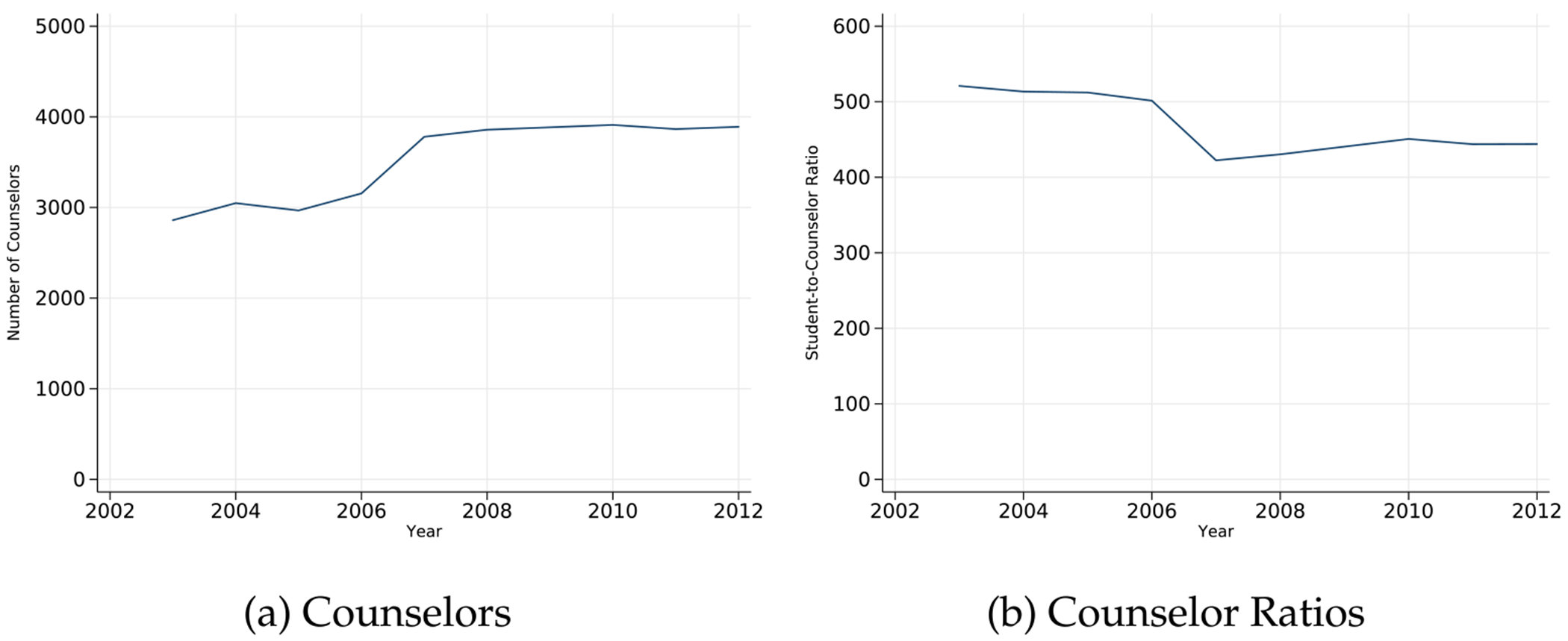
Trends in school counselors. Notes: Figures display (a) trends in the total number of counselors employed at California high schools in our analytic sample and (b) average student-to-counselor ratios from 2003–2012.

Overall, the increase in spending was associated with an increase of roughly 1000 counselors in California and a reduction of student-to-counselor ratios by nearly 100 students per counselor.^13^ Per Fig. 2, the number of counselors stagnated after program termination, and the number of students per counselor increased.

**Fig. 3.**
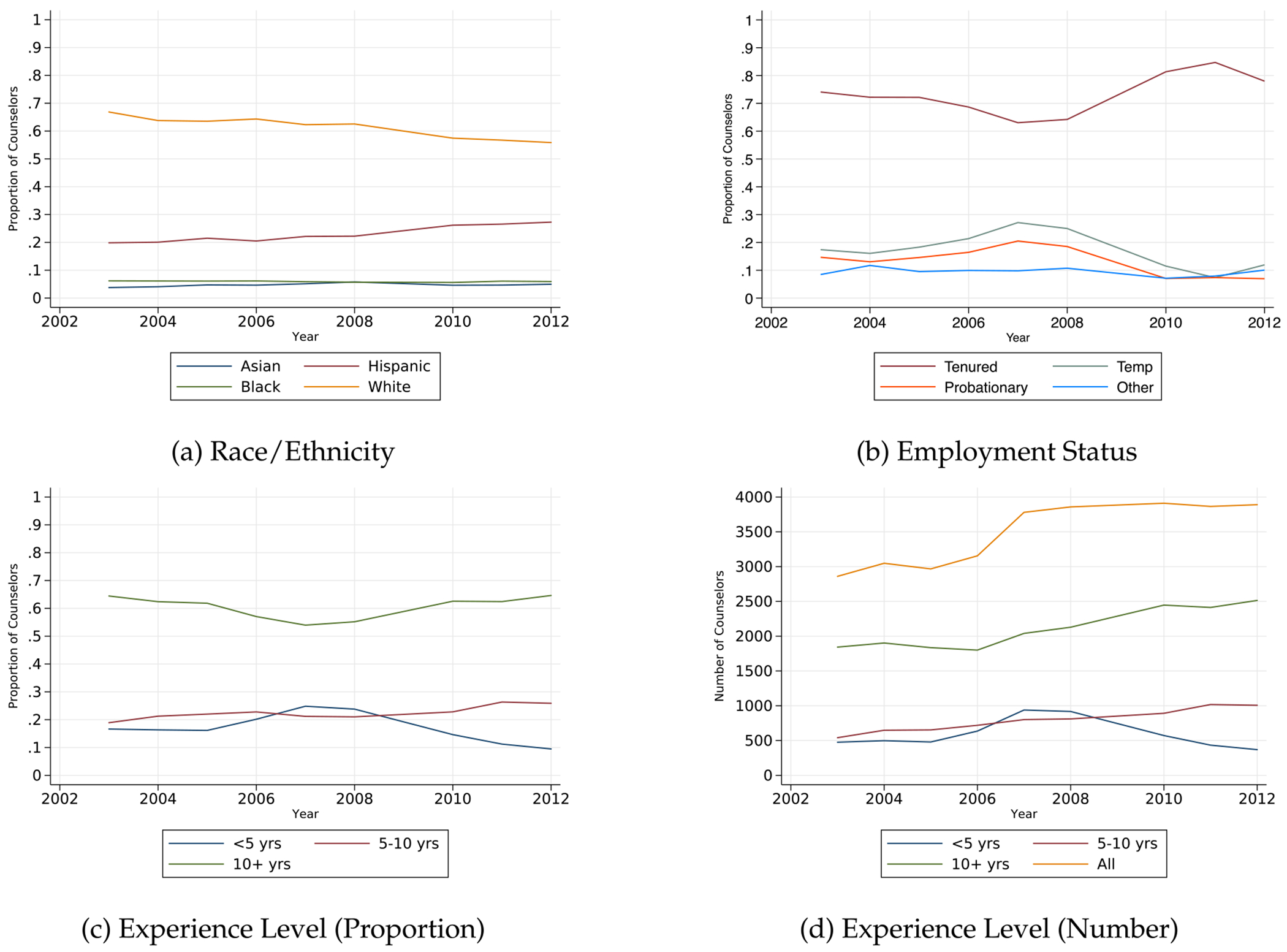
Trends in counselor characteristics. Notes: Figures display trends in counselor employment and demographic characteristics from 2003–2012. Panels (c) and (d) show the proportion and total number of counselors by experience level, respectively, as broken down by whether schools reported counselors as having less than 5, 5 to 10, or more than 10 years of counseling experience.

The initial increase in counselor hiring also led to a shift in the demographics and employment characteristics of school counselors. Fig. 3 shows that counselors hired during SSCP program years were slightly more likely to be Hispanic and less likely to be white.^14^ In addition, the proportion of tenured counselors declined from 70 percent of all counselors in 2005 to about 60 percent in 2007. Declines in the proportion of tenured counselors coincided with increases in the proportion of temp and probationary counselors. Counselors with less than 5 years of experience were most likely to be hired during SSCP program years and average counselor experience declined. We explore the potential repercussions of changes in counselor experience further in the discussion section along with policy implications for the counselor labor market.

We also explored how these patterns varied by school characteristics. Table A.17 shows how the proportion of non-white, male, temporary and least experienced counselors changed from 2005 through 2008 for schools that used SSCP funds and whether trends varied by urbanicity and the share of students who were eligible for free or reduced price lunch (FRPL). Schools in urban areas or with more than 50 percent of students receiving FRPL had a higher proportion of nonwhite counselors. At high FRPL schools, the proportion of non-white counselors decreased (indicating these schools disproportionately hired white counselors) while the share of non-white counselors increased at urban schools. The proportion of temporary and least experienced counselors increased across all school types, nor does it appear that less experienced counselors were disproportionately hired in certain types of schools.

Finally, we examined whether other types of funding changed at this time. We found no evidence of schools changing staffing or spending levels for other types of positions (e.g., social workers or psychologists). In addition, the SSCP did not appear to crowd out or crowd in spending on school counselors, suggesting funds were used in accordance with the policy (see Table A.18).

### 5.2. Main results

First, we present our event study results for outcomes where joint significance tests in the pre-period are not statistically significantly different from zero (see Tables 3 & 4). Then, we show results for a few additional outcomes where estimates may be impacted by pre-trends in outcomes.

**Fig. 4.**
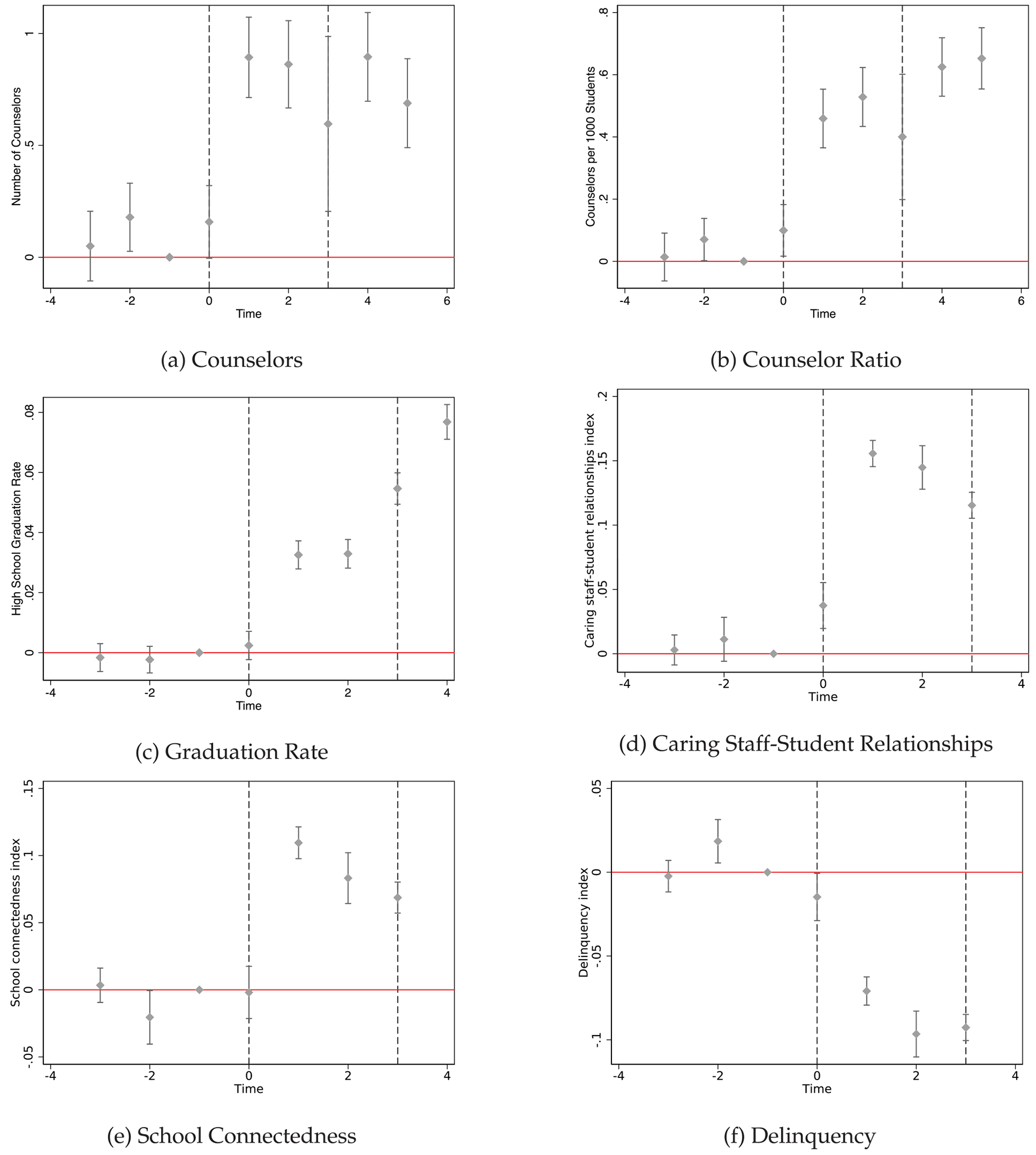
SSCP effects on counselors, high school graduation & school climate. Notes: Panels show event study estimates as specified in Eq. (2). Panel (a) shows effects on the number of high school counselors and (b) shows effects on counselor ratios (counselors per 1000 students) for schools in our analytic sample. High school graduation rate is staggered by 2 years; for instance, the graduation rate for 2006 reports on the graduation rate for students who were sophomores in 2006, since this is the year they first took the high school exit exam. Coefficients in panels (d), (e), and (f) are reported in standard deviations. Time 0 coincides with the first year a school received SSCP funds.

Fig. 4 shows the average number of counselors per high school and counselors per 1000 students increased with the implementation of SSCP. Time zero indicates the year in which the school first received SSCP funds. Changes in the second and third years (time 1 and time 2) in which a school received SSCP funds were larger than the initial year of policy implementation, which suggests that it may have taken time for schools to find counselors to hire. This is consistent with the patterns in Fig. 2. In 2009, schools were no longer required to spend SSCP funds on school counselors, which may explain the stagnating number of counselors in later years. Table 3 indicates that the average high school hired roughly one additional counselor, and the ratio of counselors per 1000 students increased by .578.^15^ Estimated effects of SSCP on the number of counselors and the counselor ratio are robust across models with and without controls.

Remaining columns in Table 3 and graphs in Fig. 4 show the policy effects on high school graduation rates, exit exam pass rates, and school climate indices for caring-staff student relationships. SSCP led to a three percentage point increase in high school graduation rates, with more pronounced effects several years after policy implementation. Observed increases in the overall CAHSEE pass rate (among 10th graders) may contribute to observed increases in high school graduation. In the models with controls, we see a small increases of half a percentage point in exit exam pass rates.^16^ We also find that the SSCP led to large increases of 0.15 standard deviations in reports of caring-staff student relationships.

**Fig. 5.**
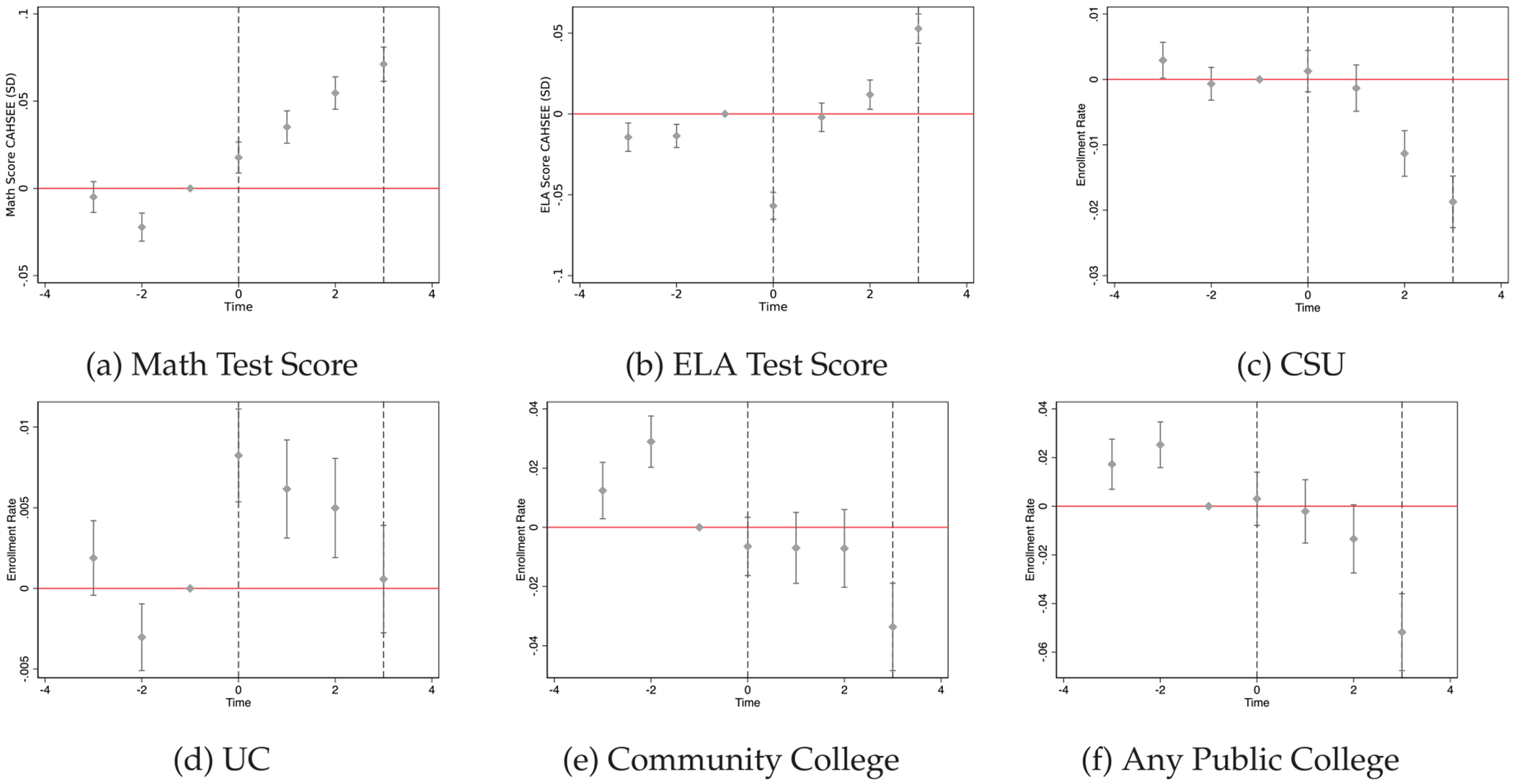
SSCP effects on exam scores and college attendance. Notes: Panels show event study estimates as specified in Eq. (2). Estimates in panels (a) and (b) are in standard deviations. Effects on college enrollment outcomes include enrollment in community college, California State Universities, University of California schools, or any California public college. Time 0 coincides with the first year a school received SSCP funds.

We also see some evidence in Fig. 5 that the policy increased school connectedness by approximately 0.11 standard deviations and reduced delinquency by 0.081 standard deviations. The models for school connectedness are close to failing the pre-trends test, though patterns in Fig. 5 do not indicate a strong pre-trend and also indicate a large break in trends when the policy started. The estimates for delinquency fail conventional pre-trends tests but, again, the shift in outcomes is much larger than the apparent direction of trends. The appendix also shows estimates for specific survey questions related to students’ sense of belonging and whether they feel supported and safe at school. For instance, Table A.16 highlights that students were more likely to report an adult at school caring about them, that an adult told them they did a good job, that an adult at school listens to them, or that they feel safe at school after the SSCP was implemented. These results suggest that counselors had a substantial impact on improving student perceptions of school climate, which in turn may contribute to observed improvements in academic achievement and college access.

Our main models for exam scores on the math and English Language Arts (ELA) portions of the CAHSEE exam as well as college outcomes are presented in Table 4 and Fig. 5. Each of these outcomes fails pre-trend tests for joint significance, suggesting confounding trends in the pre-period may bias observed effects of SSCP. Nevertheless, these results still offer descriptive insights into the effects of SSCP on these outcomes.

Table 4 also shows that exit exam math scores increased by roughly 0.05 standard deviations, and ELA scores increased by 0.01 standard deviations. Exit exams were a key barrier to high school graduation up until 2015, when California passed legislation to remove the exit exam as a requirement for high school graduation. While the simultaneous implementation of high school exit exam requirements could potentially lead to improvements in student achievement (e.g., by motivating students), Reardon, Arshan, Atteberry, and Kurlaender (2010) found that California’s exit exam requirement for graduation did not lead to meaningful changes in course-taking, achievement or high school graduation for students near the passing threshold. Accordingly, it is unlikely that the change in CAHSEE requirements in 2006 drives the positive impacts we document. If anything, it may bias our estimates downward.

**Fig. A.4.**
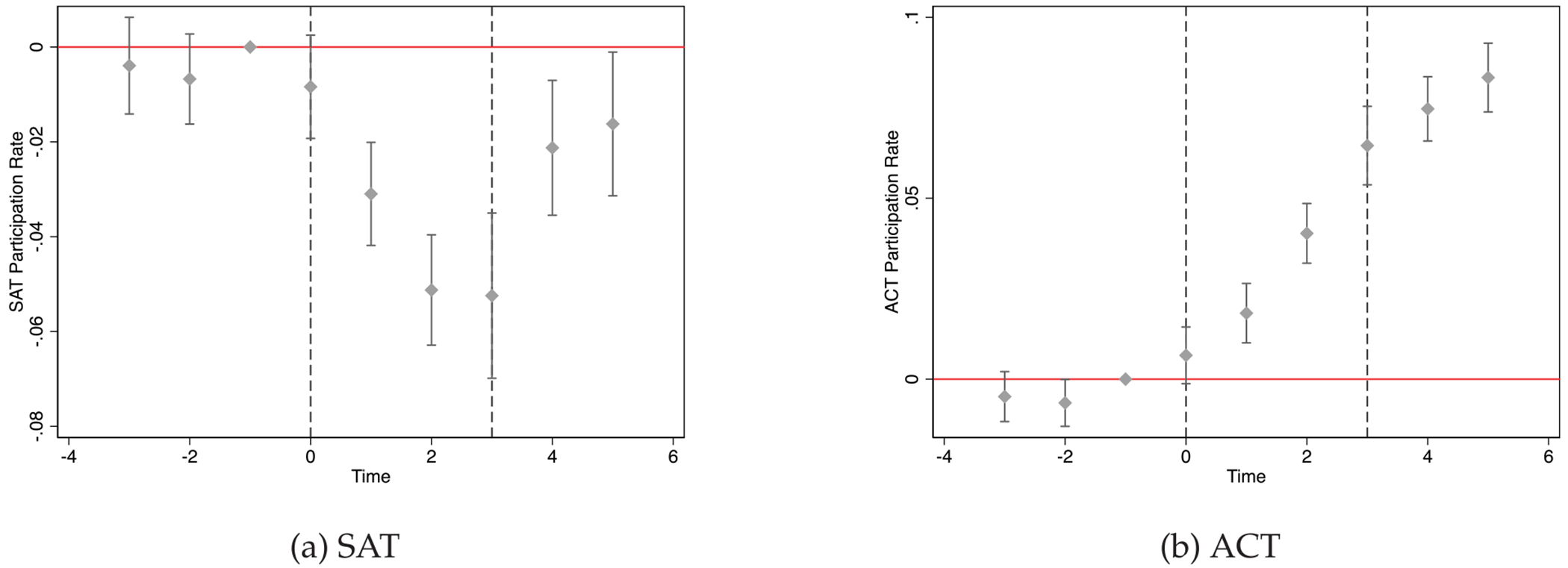
Effects on SAT & ACT test-taking. Notes: Panels show event study estimates as specified in Eq. (2). Estimates in panels (a) and (b) correspond to the change in the percentage of students who took the SAT or ACT at schools in our analytic sample.

There is suggestive evidence that University of California (UC) enrollment increased by roughly 1 percentage point after SSCP implementation, while estimates for California State University (CSU) enrollment are negative and may offset observed gains in UC enrollment. While the estimates fail the conventional pre-trends test, Fig. 5 shows a notable trend break for UC enrollment, suggesting patterns are unlikely to be fully driven by pre-trends. Increases in UC enrollment, and concurrent declines in CSU enrollment, may indicate the potential for counselors to alter college decision-making among inframarginal college-going students and increase the selectivity of colleges these students attend.^17^ We revisit this in the Discussion. Finally, Fig. A.4 indicates that while SAT taking declined over the treatment period (which coincided with SAT test changes) ACT test-taking more than offset those declines.

**Fig. A.5.**
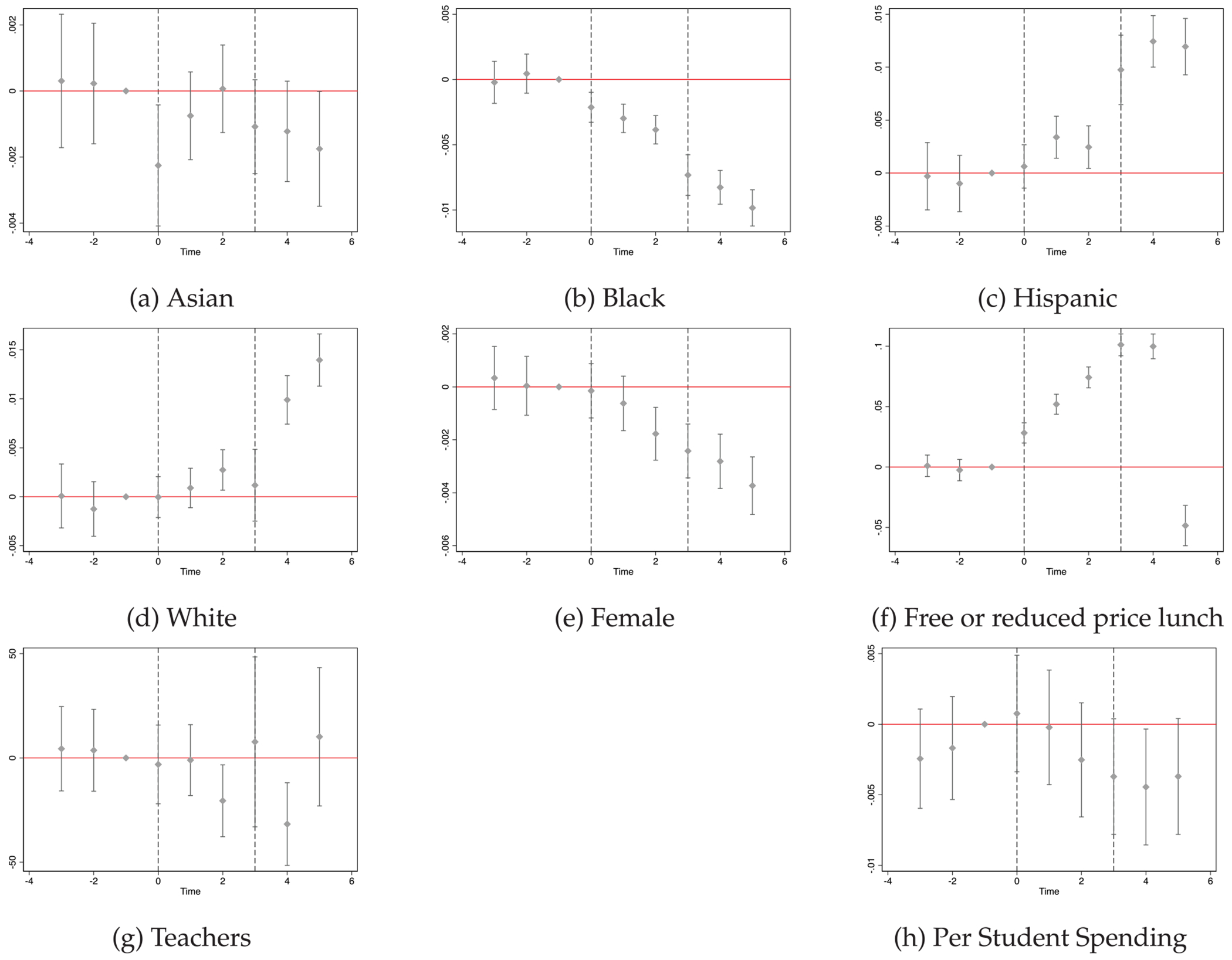
Event study results: school demographic, teacher, and spending changes. Notes: Event study plots show coefficients from Eq. (2) using linear time trends and holding treatment timing constant. In this model, 2006 is the first year of treatment for all schools that used SSCP funds. Outcomes include the share of Asian, Black, Hispanic, white, female, and students eligible for free or reduced price lunch; we also capture year over year changes in the number of teachers on staff and per student spending (in millions).

While the first-stage effect of the policy on the number of counselors on staff is clear, we may be worried that factors beyond counselors are driving observed effects on academic and school climate outcomes. Fig. A.5 shows event study results for a series of school demographics and inputs that should be unaffected by SSCP. We do observe postpolicy changes in student race and gender, but these changes are very small and ambiguous as to their relationship to observed treatment effects given baseline academic and school climate outcomes. For instance, after SSCP was implemented we see less than a half a percentage point decline in the share of Black students and female students and increases of a similar magnitude in the share of Hispanic and white students. The share of students eligible for free or reduced price lunch increased, but these increases are indicative of national trends in increased participation in the National School Lunch Program and changes in access to the program (Toossi, Todd, Guthrie, & Ollinger, 2004). Importantly, we see no evidence of changes in the number of teachers on staff or per-student spending exclusive of SSCP spending. The lack of changes to teacher staffing, classroom ratios, and non-SSCP spending bolster our claims that SSCP’s expansion of funding for counselors is driving observed effects on student outcomes.

### 5.3. Heterogeneity across students and schools

Next, we explore variation in our estimates across student and school characteristics. This is important because counselors may focus their time on serving certain types of students, some schools may have an easier time hiring qualified counselors than others, and schools may benefit more from counselors based on certain characteristics. We first look at variation by student characteristics for the outcomes we have at the student level. Table 5 shows that impacts on the CAHSEE exam scores and pass rates were positive for all groups, but larger for men, Black or Hispanic students and students from more socioeconomically disadvantaged families than their peers. Black, Hispanic and socioeconomically disadvantaged students are roughly 20 percentage points less likely to pass the CAHSEE exam than their peers, so improving their performance holds important equity implications. For most outcomes, differences by gender, race/ethnicity and socioeconomic disadvantage are statistically significant at the one percent level.^18^

The three columns on the right of Table 5 also show differences in the school climate outcomes by gender and race/ethnicity. It indicates that impacts are significantly larger for men than women when looking at caring staff–student relationships and delinquency. In addition, impacts are significantly larger for Black or Hispanic students relative to white or Asian students when looking at caring staff–student relationships and school connectedness. Improvements in school climate may explain some of the observed changes in academic achievement for these groups.

Second, we look at variation in outcomes by school characteristics. The first two columns of Table 6 show that changes in counselor caseloads are comparable across high and low poverty schools and urban and rural schools. Large schools experienced larger gains in the number of counselors whereas changes in the counselor ratios per 1000 students are more comparable across school size categories.^19^ Improvements in high school graduation are comparable across all school subgroups.

Effects on college enrollment are larger at high poverty schools and in rural areas. In particular, SSCP is associated with a 1 percentage point increase in UC enrollment at high poverty schools and a 5 percentage point increase in college enrollment in rural schools. There is no significant impact on college enrollment rates in low poverty schools, where we see that the policy had more of an impact on the types of colleges that students attended: the policy led to a reduction in attendance at CSU schools but a similar magnitude increase in attendance at UC schools. Thus, counselors may be important for helping students select and attend highly selective schools, which can be beneficial for future outcomes including degree attainment and earnings (Bleemer, 2022; Cohodes & Goodman, 2014). Panel (C) also indicates that the program had larger effects on college enrollment among students in large high schools, though overall effects on public college going are not significant. This could be because the policy had larger impacts on access to school counselors in these schools.

We present similar results on CAHSEE achievement and school climate outcomes by school characteristics in Table 7. Findings indicate that SSCP led to significantly larger increases in CAHSEE exam pass rates and scores in high poverty schools relative to low poverty schools. In high poverty schools, SSCP is associated with an overall increase in pass rates of 3.1 percentage points and score increases of 0.09 standard deviations for math and 0.05 for ELA. Impacts on pass rates are also marginally higher in large schools relative to small schools. Indices for caring staff–student relationships are higher (by 0.02 standard deviations) in high poverty schools relative to low poverty schools while effects on school connectedness and delinquency are comparable across school poverty levels. The effects on all three of these indices are also higher for rural schools relative to urban schools.

Finally, we look at variation in outcomes based on the characteristics of counselors that schools hired. We hypothesized that hiring more experienced counselors could lead to positive impacts while hiring inexperienced counselors could lead to worse student outcomes. Table A.12 reports results by whether the average level of experience in a school increased, decreased, or stayed the same with the implementation of SSCP. There are no clear patterns to suggest that the policy impacts were driven by the types of counselors hired. This aligns with prior research from Mulhern (2023), which found that counselor experience was not a strong predictor of student outcomes. The lack of clear effects of counselor experience levels on student outcomes may speak to the policy’s requirement to hire credentialed counselors, leaving unchanged the pre-policy licensure requirements; it may also intimate that the benefits of hiring an additional counselor regardless of experience level may be higher than increasing counselor experience in settings where baseline access to counselors is low.

We also examine whether alignment between counselors’ identified race/ethnicity and the racial composition of schools impacts outcomes given well-documented effects of teacher–student race match in improving student achievement (e.g. Dee, 2005; Egalite, Kisida, &Winters, 2015). Table A.15 highlights results from a similar event study framework that captures the effects of hiring a counselor post-SSCP whose race/ethnicity is similar to that of the high school. For instance, row 1 indicates the effects of hiring a counselor who identified as Asian at a high school with above-median Asian student representation. Results suggest that schools serving an above-median share of Hispanic students and that hired an Hispanic counselor saw increases of about 2 percentage points in high school graduation and the share of public college enrollment, driven by increases in community college and UC enrollment. Schools serving above median Black and white students and who hired an additional Black and white counselor, respectively, saw smaller but significant increases in high school graduation, whereas effects on college enrollment were more muted.

### 5.4. Robustness checks

**Fig. A.6.**
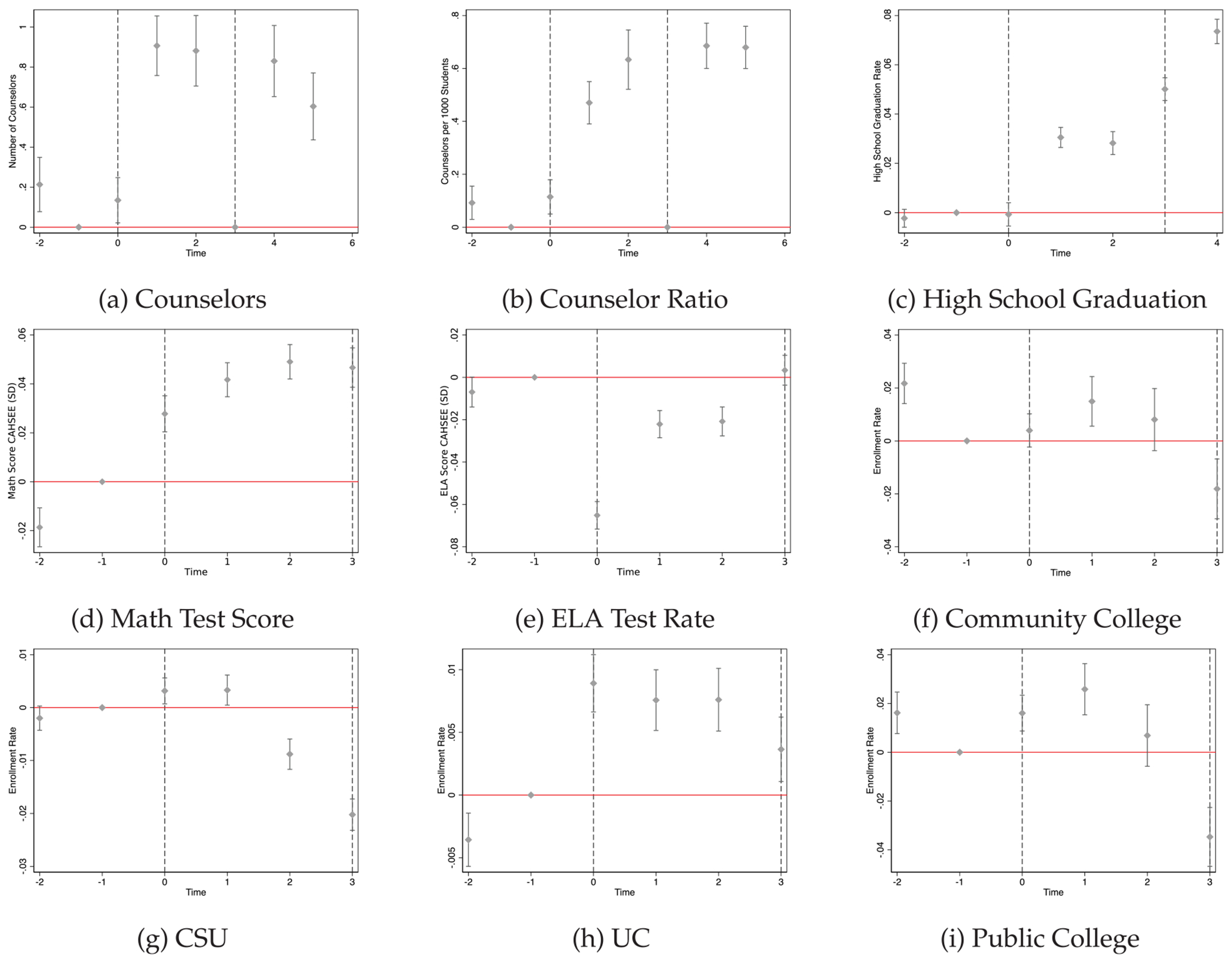
Intent to treat estimates. Notes: Event study plots show coefficients from Eq. (2) using linear time trends and holding treatment timing constant. In this model, 2006 is the first year of treatment for all schools that used SSCP funds. The ELA and math pass rates are based on student-level data and all other estimates are from school-level models.

The appendix contains additional robustness checks for our main estimates. First, we show that patterns are similar when we cluster standard errors by school or by district instead of at the school by year level (see Table A.5). Second, we fit intent to treat models where treatment is defined as the first year of the SSCP (2006) rather than the first year each school received funds. Schools that did not receive funding are included in these estimates and also considered treated in 2006. These results are in Tables A.7, A.8, and Fig. A.6. They are comparable to our main effect estimates, which is not surprising given high take-up rates across districts in initial program years. Third, we fit models which include our first year of treatment in the average treatment effect. Tables A.9 and A.10 contain these results. The magnitudes are slightly smaller (because the policy had smaller effects in its first year), but the patterns are qualitatively similar.

Fourth, we use Callaway & Sant’Anna’s semi-parametric DID estimator that allows for treatment timing to vary. This model also accounts for year-specific variation more flexibly than our linear time trends model.^20^ This model then compares outcomes before and after receipt of SSCP funds for high schools that participated in the program versus those that did not. In contrast to our main results from event studies controlling for linear time trends in the pre-period, the sample for this TWFE approach includes high schools that never received SSCP funds. As mentioned previously, schools that never received SSCP funds tended to enroll fewer students and were more likely to be located in rural areas. They were also relatively few: less than 10 percent of high schools in the sample never received SSCP funds. As is to be expected given the small size of the counterfactual group, results from this approach are less precise than our main estimates. And while few outcomes of interest had pre-treatment averages that were significantly different from zero, chi-squared values from joint significance tests in the pre-period indicate that many outcomes do not satisfy the parallel trends assumption.

**Fig. A.7.**
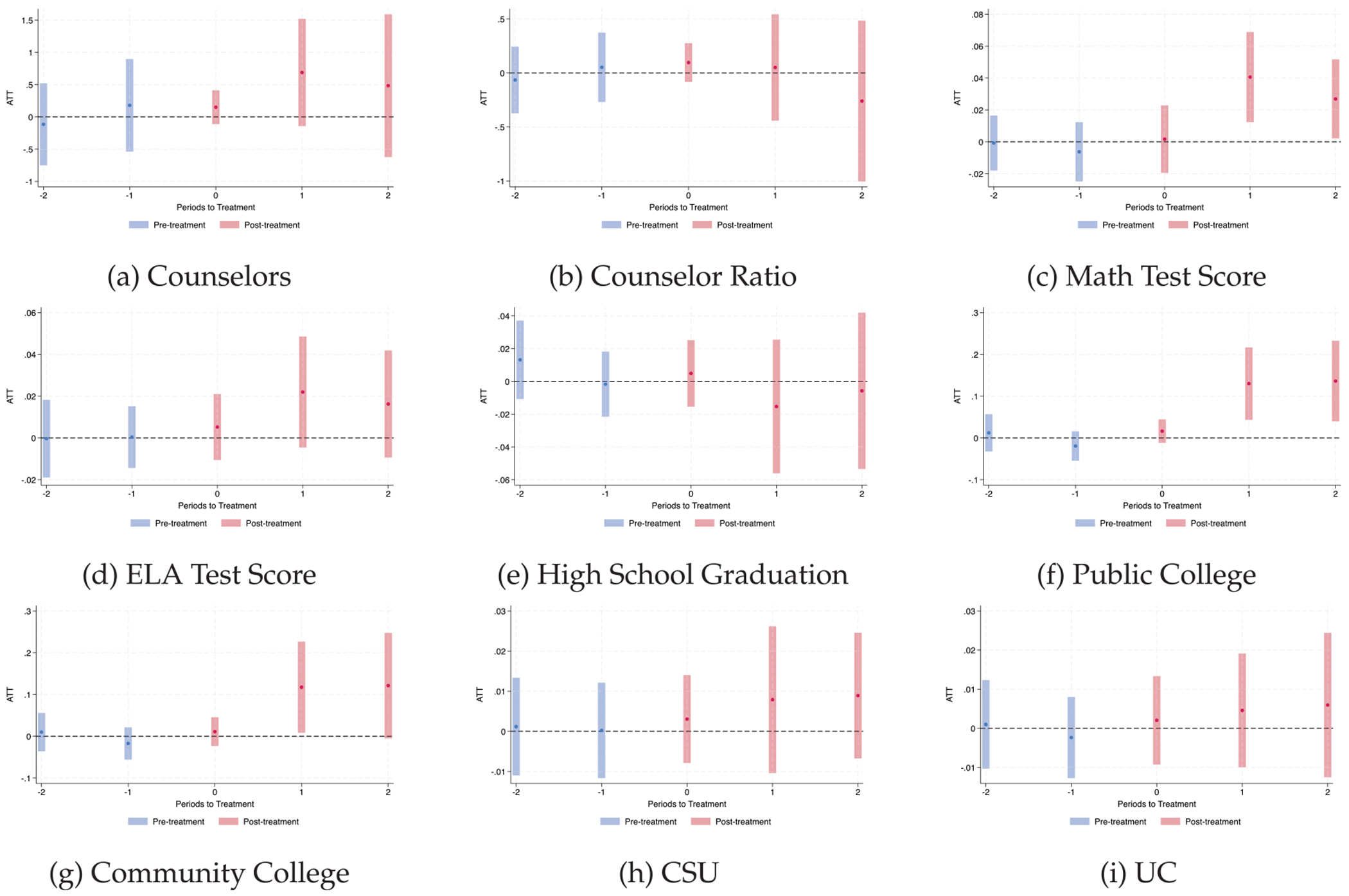
Estimates from Callaway and Sant’Anna approach. Notes: Event study plots show coefficients from CSDID models for the first group of policy adopters in 2006 relative to not yet treated schools.

With these considerations in mind, results should be interpreted descriptively but do affirm previously reported findings that SSCP increased the number of counselors on staff, though point estimates attenuate to .457 compared to the 1 additional counselor we observed in our main models (see Table A.11 & Fig. A.7). TWFE estimates on counselor ratios attenuate toward zero and are not significant. These findings appear to be driven by two phenomena: schools that did not receive SSCP funds still increased the number of counselors on staff, albeit not at the same rate as those that did, and experienced sharper declines in student enrollment over the years included in our analysis. Furthermore, effects on high school graduation attenuate toward zero, and increases in public college enrollment are much larger and driven by substantial increases in community college enrollment. We cannot rule out null effects on CAHSEE achievement outcomes or school climate measures.

We also look at how the amount of SSCP funding a school received is related to student outcomes. For this, we fit the same type of event study models as in our main models but interact the treatment indicator with SSCP funds received (in terms of $100,000 dollars). These estimates (see Tables A.13 & A.14) mirror those from our main models, indicating that SSCP funds increase the number of counselors, public college going, test scores, pass rates, and school climate while reducing delinquency.

Last, to address some of the aforementioned issues with our panel-based estimators, we use an instrumental variables approach to estimate effects of SSCP on outcomes, where we use student SSCP spending as an instrument for counselor to student ratios (see Table A.6). Models control for school demographic characteristics and are limited to the 2007–2008 academic years post policy implementation. Results are mostly imprecise, but suggest SSCP had no effect on high school graduation rates but increased public college going upwards of 10 percentage points. These increases are mostly driven by increased community college and CSU enrollment. Results from the IV approach are very noisy for the high school exit exams and school climate measures.

## 6. Discussion

Our results indicate that the SSCP increased spending on counselors and the number of counselors on staff, which in turn reduced student-to-counselor ratios. These changes are associated with a three percentage point increase in high school graduation rates, small improvements in math and ELA test scores and public college attendance, and more than a 0.1 standard deviation improvement in school climate indices. These results align with some of the prior literature evaluating the effects of school finance reforms and counselors more specifically. For example, Lafortune et al. (2018) found that school finance reforms increased student achievement as captured through test scores by 0.1 standard deviations and narrowed the gap between low- and high-income school districts; Hurwitz and Howell (2014) found that increased access to counselors and reductions in student-to-counselor ratios improved four-year college enrollment by 10 percentage points; and Reback (2010) found that counselors helped to reduce school climate outcomes such as suspensions and weapon-related incidents by 12 percentage points, albeit this study focused on elementary rather than high schools.

Increased access to counselors may also shift the types of colleges that students attend. Our main and instrumental variable models and robustness checks suggest counselors may induce inframarginal college-goers to enroll in more selective institutions and marginal college-goers to enroll in community college. Impacts on academic and school climate outcomes were larger in high poverty schools and rural schools and for students who were socioeconomically disadvantaged, male, or students who were Black or Hispanic. Thus, counselors may help to reduce existing equity gaps in academic achievement and college enrollment. Despite the policy leading to a large influx of less experienced counselors, we do not find any consistent evidence that policy impacts varied based on the experience levels of the counselors hired during the program. In contrast, we do offer suggestive evidence that the relationship between student and counselor demographics may influence outcomes.

We do not see any evidence that the program crowded out or crowded in spending on school counselors. Per policy guidelines, SSCP funds were required to supplement rather than supplant counselor spending. Treatment effect estimates may be biased toward zero if SSCP funds crowded out counselor spending or, conversely, we might see larger effects for some schools if SSCP funding crowded in additional counselor spending. To assess this, we regress total counselor spending on SSCP spending, including district fixed effects and limiting to program years. We find that districts spent $.958 dollars on guidance counseling services for every $1 of SSCP money spent (see appendix Table A.18). This suggests schools followed policy guidelines and that SSCP funds did not crowd out (or in) overall counselor spending.

While the SSCP led to a significant change in the number of school counselors in California and had a meaningful impact on student outcomes, the changes still fell short of reducing counselor ratios to the American School Counselor Association’s recommendation of 250 to 1. It is possible that larger reductions in caseloads may be needed to observe larger benefits. In addition, tradeoffs between increasing access to counselors and reducing average counselor experience may be different in a state with lower starting student to counselor ratios. California had one of the highest student to counselor ratios at the time (≈ 500:1), and it continues to have a ratio well above the national average (464:1 relative to the average of 385:1) (American School Counselors Association, 2023).

**Fig. A.8.**
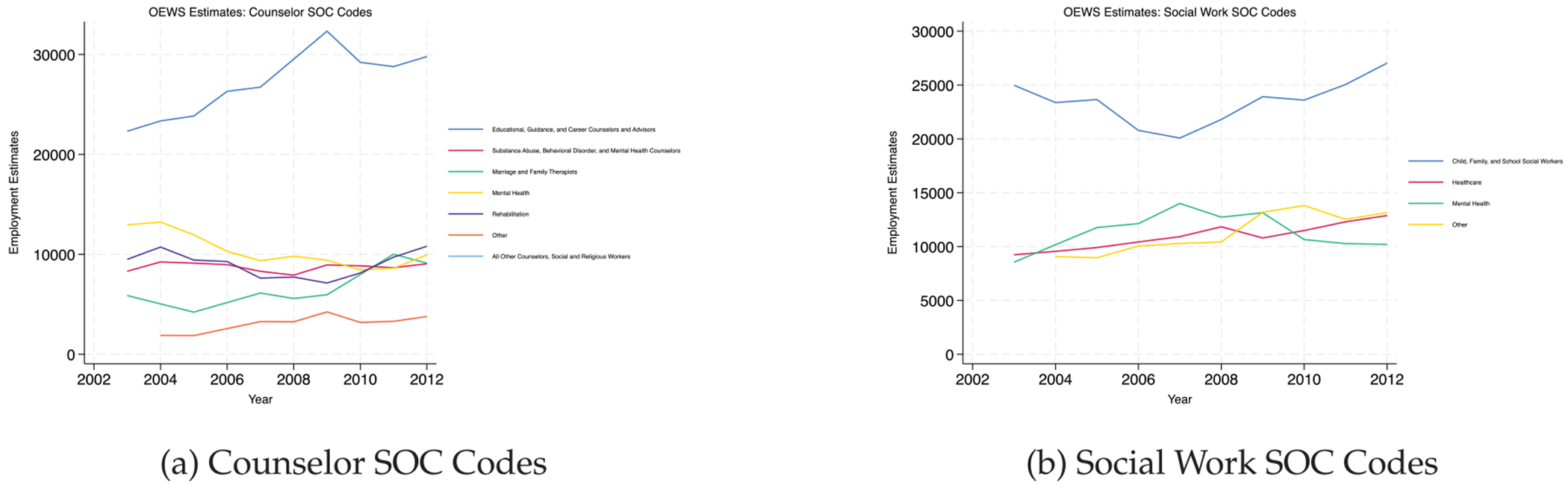
CA employment estimates for relevant occupations. Notes: These figures show the number of people employed in each counselor and social work occupation in California by year.

Impacts of similar policies may vary across state contexts based on their labor markets and pool of potential counselors. We examined where schools may have found counselors to hire amid this rapid expansion and where these counselors may have gone when the policy ended using data from Occupational Employment and Wage Statistics (OEWS) on employment rates in California. Fig. A.8 shows these results. The number of mental health counselors declined during the SSCP years, so schools may have hired school counselors away from this field. Similarly, it appears that counselors let go by schools after SSCP funding was terminated may have gone into different fields of social work, particularly child, family, and school social work.^21^ Our data limit the extent to which we can further analyze observed phenomena in the counselor labor market. For example, the lack of a unique, year-over-year staff identifier prevents us from exploring counselor turnover and poaching. Future research should assess whether more experienced counselors were poached by higher-resourced schools and whether the policy had an impact on within-school counselor turnover.

Furthermore, program impacts may have varied if SSCP had been implemented for a longer period of time. Program funding only existed for three years before being rolled into general education budgets in 2009 as a result of the Great Recession. Effects on students’ academic and social-emotional outcomes may be larger with more sustained and consistent program funding, especially for outcomes such as high school graduation and college enrollment which can be impacted by multiple years of exposure to a counselor.

Finally, it is important to consider the schools that did not use SSCP funds. The relatively few districts that did not use SSCP dollars were concentrated in rural areas with lower student enrollment and counselor ratios. In these districts, the amount of money allotted based on enrollment may not have been enough to hire a full time counselor, and these districts may have faced additional hurdles in recruiting and hiring licensed counselors. In addition to concerns around counselor experience, this suggests the importance of pairing increased funding for school counselors with policies that support and ensure adequate counselor labor supply.

While this study provides insight into how statewide policies to expand school counseling may influence student outcomes, there are a few additional questions that will be important to examine in the future. Here, we focus exclusively on outcomes in and shortly after high school, though it would be valuable to know how large-scale expansions of counseling earlier on may impact long-term academic and school climate outcomes. In addition, the college enrollment outcomes we are able to measure omit private and out-of-state colleges; this accounts for only 10 percent of California high school graduates, but inclusion of these students may add more clarity to counselor effects on college-going. Last, we are limited in our ability to detect spillover effects on teachers, who may take on some counseling responsibilities in the absence of sufficient counselors.

## 7. Conclusion

This paper presents some of the first evidence on the impacts of a large scale program to expand school counseling. The additional funds that California provided to middle and high schools as part of the Supplemental School Counseling Program led to increases in the number of counselors in these schools and a reduction in the number of students assigned to each counselor. The expansion of school counseling pulled many newer and less experienced counselors into schools, suggesting that the supply of counselors is important to consider before implementing large programs like this. Importantly, the program significantly improved high school graduation and may have improved public college-going. Policy effects on student perceptions of school climate highlight the critical role that counselors play in supporting students’ academic outcomes, social-emotional health, and school climate at-large. The program also had larger impacts in high poverty and rural schools, and for men, more socioeconomically disadvantaged students as well as Black and Hispanic students. As policymakers and school officials continue to grapple with mental health crises and equity gaps in academic achievement and college access post-Covid 19, counselors may be a valuable resource for students and families.

## Figures and Tables

**Table 1:** Sample high school characteristics.

Characteristics	Enrollment (1)	Female (2)	Asian (3)	Black (4)	Hispanic (5)	White (6)	Share FRPL (7)
2003	1328	0.48	0.07	0.07	0.37	0.42	0.35
2004	1326	0.48	0.07	0.08	0.38	0.41	0.35
2005	1319	0.48	0.07	0.08	0.39	0.39	0.39
2006	1302	0.48	0.07	0.07	0.40	0.38	0.39
2007	1290	0.48	0.07	0.07	0.41	0.37	0.43
2008	1267	0.48	0.07	0.07	0.42	0.35	0.45
2009	1256	0.48	0.07	0.07	0.44	0.34	0.49
2010	1234	0.48	0.07	0.07	0.46	0.33	0.48
2011	1218	0.47	0.07	0.07	0.47	0.32	0.39
2012	1196	0.47	0.07	0.07	0.48	0.31	0.54
Outcomes	Graduation Rate (1)	ELA (2)	Math (3)	Community College Enrollment (4)	CSU Enrollment (5)	UC Enrollment (6)	Public College Enrollment (7)

2003	0.90	0.68	0.66	0.28	0.09	0.08	0.45
2004	0.90	0.69	0.66	0.30	0.10	0.07	0.47
2005	0.88	0.69	0.68	0.27	0.11	0.07	0.45
2006	0.88	0.69	0.68	0.27	0.12	0.08	0.47
2007	0.88	0.71	0.69	0.28	0.12	0.08	0.48
2008	0.86	0.71	0.70	0.28	0.12	0.07	0.47
2009	0.88	0.72	0.71	0.26	0.12	0.07	0.45
2010	0.85	0.80	0.79	.	.	.	.
2011	0.86	0.73	0.72	.	.	.	.
2012	0.89	0.73	0.73	.	.	.	.

Notes: Reported school characteristics include average high school enrollment, gender and racial composition of the student body, and the proportion of students who are eligible for free or reduced price lunch. Reported outcomes include average four-year high school graduation rate and the California High School Exit Exam (CAHSEE) pass rates for grade 10 students in English Language Arts and Math. Community College, CSU, and UC share refers to the proportion of students who enrolled in a California public community college, California State University, and University of California school, respectively. Public college share captures the percent of students who enrolled in any California public college.

**Table 2:** Sample high school characteristics, by SSCP takeup.

	Used SSCP funds (1)	Never used SSCP funds (2)	Difference in means (3)	P-value (4)
Demographic Characteristics				
Share FRPL	0.424	0.435	0.011	0.705
Female	0.477	0.479	0.002	0.773
Asian	0.071	0.028	−0.043	0.001
Hispanic	0.418	0.296	−0.122	0.000
Black	0.078	0.044	−0.033	0.006
White	0.359	0.521	0.162	0.000
Rural	0.190	0.506	0.316	0.000
Urban	0.337	0.416	0.078	0.159
High School Outcomes				
Graduation Rate	0.874	0.855	−0.018	0.227
SAT	0.310	0.251	−0.059	0.051
ACT	0.143	0.141	−0.002	0.907
ELA	0.692	0.733	0.040	0.180
Math	0.683	0.708	0.025	0.431
Community College Enrollment	0.278	0.169	−0.109	0.000
CSU Enrollment	0.123	0.074	−0.049	0.000
UC Enrollment	0.077	0.042	−0.036	0.000
Public College Enrollment	0.479	0.285	−0.194	0.000
Counselor Characteristics				
Counselor Ratio	2.486	2.534	0.049	0.885
Counselors	2.732	1.403	−1.330	0.000
0–5 Years of Experience	0.634	0.320	−0.313	0.001
5–10 Years of Experience	0.595	0.232	−0.364	0.000
10+ Years of Experience	1.503	0.851	−0.653	0.001
N	1286	77		

Notes: Data are limited to high schools in our analytic sample. We report the average high school demographic characteristics, outcomes, and counselor characteristics for high schools in districts that ever or never received SSCP funds between 2006 and 2008. P-values are reported from a ttest of the difference in means between high schools in districts that received any SSCP funding between 2006 and 2008 versus those in districts that never received SSCP funding. Counselor characteristics related to years of experience indicate the average number of counselors per school within each experience band. Counselor ratio indicates the number of counselors per 1000 students.

**Table 3:** Effects of SSCP on counselor, academic & school climate outcomes.

	Total counselors (1)	Counselor ratio (2)	High school graduation (3)	CAHSEE pass rate (4)	Caring staff-student relationships (5)	School connectedness (6)	Delinquency (7)
(A) With Controls							
SSCP	0.990*** (0.096)	0.578*** (0.047)	0.030*** (0.003)	0.005*** (0.002)	0.153*** (0.006)	0.109*** (0.007)	−0.081*** (0.005)
F-statistic	1.97	1.93	1.10	.681	1.62	2.13*	5.27***
(B) Without Controls							
SSCP	0.908*** (0.051)	0.537*** (0.023)	0.030*** (0.001)	0.001 (0.001)	0.152*** (0.003)	0.109*** (.0004)	−0.080*** (0.003)
F-statistic	1.86	1.94	.795	.248	1.66	2.09*	5.24***
N	5802	5802	5776	1,850,014	983,431	991,790	1,004,899

Notes: Heteroskedasticity robust standard errors clustered by school and year are in parentheses.

* (p < .10

** p < .05

*** p < .01).

All regressions include school fixed effects and control for linear time trends in the preperiod, or years prior to policy implementation in 2006. Estimates in panel (A) are based on regressions which control for instructional spending levels at the district, the share of students receiving free or reduced-price lunch in the school, student gender, race/ethnicity, and socioeconomic disadvantage, as well as indicators for whether the school is in a rural or urban area. Estimates in panel (B) do not include these controls. Overall pass rate refers to passing both the math and ELA exams. Pass rates are only based on students who take the test in 10th grade. These estimates are based on 2003–2008 and treatment is determined by when the school first received SSCP funds. The first year of treatment is excluded from the estimates. Estimates for the school climate outcomes in columns 5–7 are reported in standard deviations. Reported f-statistics are from a joint significance test for the period and indicate whether outcomes in the pre-period are statistically significantly different from zero.

**Table 4:** Effects of SSCP on college enrollment, academic & school climate outcomes.

	Math score (1)	ELA score (2)	CSU enrollment (3)	UC enrollment (4)	Community college enrollment (5)	Public college enrollment (6)
(A) With Controls						
SSCP	0.058*** (0.002)	0.016*** (.002)	−.006*** (0.002)	0.008*** (0.001)	−.009 (0.007)	−0.007 (0.007)
F-statistic	10.67^***^	7.76^***^	3.38**	9.84***	17.44***	11.17***
(B) Without Controls						
SSCP	0.051*** (0.002)	−0.016*** (.002)	−0.003*** (0.001)	0.006*** (0.001)	0.006** (0.003)	0.010*** (0.004)
F-statistic	8.27***	1.32	3.21**	9.60***	12.03***	5.75***
N	1,895,506	1,895,506	4267	4267	4267	4267

Notes: Heteroskedasticity robust standard errors clustered by school and year are in parentheses.

* (p < .10

** p < .05

*** p < .01).

All regressions include school fixed effects and control for linear time trends in the preperiod, or years prior to policy implementation in 2006. Estimates in panel (A) are based on regressions which control for instructional spending levels at the district, the share of students receiving free or reduced-price lunch in the school, student gender, race/ethnicity, and socioeconomic disadvantage, as well as indicators for whether the school is in a rural or urban area. Panel B excludes these controls. Math and ELA scores are reported in standard deviations and are only based on students who take the test in 10th grade. These estimates are based on 2003–2008 and treatment is determined by when the school first received SSCP funds. The first year of treatment is excluded from the estimates. Reported f-statistics are from a joint significance test for the period and indicate whether outcomes in the pre-period are statistically significantly different from zero.

**Table 5:** Impacts on high school exit exam and school climate by student characteristics.

	math scale score (1)	ELA scale score (2)	CAHSEE pass rate (3)	Caring staff–student relationships (4)	school connectedness (5)	delinquency (6)
(A) By Gender						
Male	0.069*** (0.005)	0.030*** (0.005)	0.008*** (0.002)	0.172*** (0.006)	0.109*** (0.007)	−0.099*** (0.006)
Female	0.047*** (0.005)	0.002 (0.005)	0.003 (0.002)	0.135*** (0.006)	0.108*** (0.007)	−0.063*** (0.005)
P-value Difference	0.00	0.00	0.00	0.00	0.81	0.00
Male Mean	0.50	0.31	0.73	−0.08	−0.04	0.11
Female Mean	0.46	0.56	0.77	0.03	0.00	−0.22
(B) By Race/Ethnicity						
Black/Hispanic	0.083*** (0.006)	0.052*** (0.005)	0.032*** (0.002)	0.170*** (0.007)	0.123*** (0.008)	−0.088*** (0.006)
White/Asian	0.037*** (0.005)	−0.015*** (0.005)	−0.017*** (0.002)	0.142*** (0.006)	0.100*** (0.007)	−0.079*** (0.005)
P-value Difference	0.00	0.00	0.00	0.00	0.00	0.34
Black/Hispanic Mean	0.12	0.09	0.63	−0.10	−0.11	0.03
White/Asian Mean	0.83	0.77	0.87	0.08	0.10	−0.17
(C) By FRPL Status						
Socioeconomically Disadvantaged	0.083*** (0.006)	0.051*** (0.006)	0.031*** (0.003)			
Not Socioeconomically Disadvantaged	0.045*** (0.005)	−0.002 (0.005)	−0.008*** (0.002)			
P-value Difference	0.00	0.00	0.00			
Soc. Disadv. Mean	0.17	0.07	0.63			
Not Soc. Disadv. Mean	0.67	0.66	0.82			
N	1,895,506	1,895,436	1,850,014	983,431	991,790	1,004,899

Notes: Heteroskedasticity robust standard errors clustered by school and year are in parentheses.

* (p < .10

** p < .05

*** p < .01).

All regressions include school fixed effects and control for linear time trends in the pre-period. The regressions also control for instructional spending levels at the district, the share of students receiving free or reduced-price lunch in the school, student gender, race/ethnicity, and socioeconomic disadvantage, as well as indicators for whether the school is in a rural or urban area. Overall pass rate refers to passing both the math and ELA exams. Pass rates are only based on students who take the test in 10th grade. These estimates are based on 2003–2008 and treatment is determined by when the school first received SSCP funds. The first year of treatment is excluded from the estimates. Panel (A) reports estimates separately by gender. Panel (B) reports estimates separately by race/ethnicity. Panel (C) reports estimates separately by whether or not the student is classified as socioeconomically disadvantaged; socioeconomically disadvantaged status is not available for school climate data. All panels include p-values reporting whether the coefficients for the different groups are significantly different from one another. Mean outcomes for each group are also reported. Estimates are reported in standard deviations for all outcomes besides the overall CAHSEE pass rate.

**Table 6:** Effects on counselor and academic outcomes, by school subgroups.

	Total counselors (1)	Counselor ratio (2)	High school graduation (3)	Community college enrollment (4)	CSU enrollment (5)	UC enrollment (6)	Public college enrollment (7)
(A) By Share of FRPL Students							
High Poverty	1.130*** (0.135)	0.620*** (0.062)	0.034*** (0.004)	−0.009 (0.010)	−0.001 (0.002)	0.013*** (0.002)	0.004 (0.010)
Low Poverty	0.932*** (0.099)	0.552*** (0.051)	0.029*** (0.003)	−0.010 (0.007)	−0.008*** (0.002)	0.006*** (0.002)	−0.011 (0.007)
P-value Difference	0.12	0.24	0.15	0.89	0.00	0.00	0.09
Mean: High Poverty	2.47	3.03	0.83	0.27	0.10	0.05	0.42
Mean: Low Poverty	2.79	2.36	0.90	0.28	0.12	0.09	0.49
(B) By Urbanicity							
Urban	0.927*** (0.115)	0.554*** (0.049)	0.030*** (0.003)	−0.025*** (0.007)	−0.008*** (0.002)	0.008*** (0.002)	−0.025*** (0.008)
Rural	1.302*** (0.157)	0.620*** (0.107)	0.032*** (0.004)	0.038*** (0.013)	0.005 (0.004)	0.011*** (0.003)	0.054*** (0.016)
P-value Difference	0.15	0.34	0.87	0.00	0.01	0.85	0.00
Mean: Urban	2.97	2.43	0.86	0.29	0.11	0.09	0.49
Mean: Rural	2.38	2.80	0.89	0.27	0.11	0.06	0.44
(C) By School Size							
Large	1.244*** (0.116)	0.527*** (0.050)	0.030*** (0.003)	0.003 (0.007)	−0.006*** (0.002)	0.010*** (0.002)	0.007 (0.008)
Medium	0.809*** (0.111)	0.590*** (0.058)	0.032*** (0.003)	−0.029*** (0.008)	−0.006*** (0.002)	0.005*** (0.002)	−0.030*** (0.009)
Small	0.473*** (0.096)	0.747*** (0.102)	0.029*** (0.004)	−0.012 (0.009)	0.001 (0.004)	0.008** (0.003)	−0.003 (0.011)
P-value Difference	0.00	0.05	0.67	0.00	0.39	0.00	0.00
P-value Difference	0.01	0.74	0.38	0.00	0.52	0.00	0.00
P-value Difference	0.00	0.04	0.68	0.75	0.04	0.92	0.66
Mean: Large	5.17	2.00	0.90	0.32	0.12	0.08	0.52
Mean: Medium	3.32	2.16	0.90	0.31	0.12	0.09	0.51
Mean: Small	0.90	3.20	0.84	0.20	0.09	0.05	0.34

Notes: All regressions include fixed effects for the year and school and control for school demographic characteristics and instructional spending. High poverty schools are defined as those with at least 50% of students receiving free or reduced-price lunch. Low poverty schools are those with fewer than 50% of students receiving free or reduced-price lunch. Large schools are defined as those with at least 2000 students, medium schools have 1000 to 1999 students and small schools have fewer than 1000 students. These estimates are based on 2003–2008 and treatment is determined by when the school first received SSCP funds. All panels include p-values reporting whether the coefficients for the different groups are significantly different from one another. Mean outcomes for each group are also reported. Heteroskedasticity robust standard errors clustered by school are in parentheses.

* (p < .10

** p < .05

*** p < .01).

**Table 7:** Impacts on high school exit exam and school climate by school characteristics.

	math scale score (1)	ela scale score (2)	cahsee pass rate (3)	Caring staff–student relationships (4)	school connectedness (5)	delinquency (6)
(A) By Share of FRPL Students						
High Poverty	0.092*** (0.006)	0.047*** (0.006)	0.031*** (0.003)	0.169*** (0.009)	0.109*** (0.011)	−0.083*** (0.008)
Low Poverty	0.045*** (0.005)	0.004 (0.004)	−0.004** (0.002)	0.150*** (0.006)	0.109*** (0.007)	−0.081*** (0.005)
P-value Difference	0.00	0.00	0.00	0.02	0.98	0.75
High Poverty Mean	0.21	0.14	0.65	−0.06	−0.11	−0.01
Low Poverty Mean	0.62	0.58	0.80	−0.00	0.02	−0.08
(B) By Urbanicity						
Urban	0.062*** (0.005)	0.015*** (0.005)	0.007*** (0.002)	0.145*** (0.006)	0.096*** (0.008)	−0.074*** (0.005)
Rural	0.052*** (0.009)	0.018* (0.009)	0.004 (0.004)	0.174*** (0.012)	0.145*** (0.016)	−0.107*** (0.010)
P-value Difference	0.08	0.78	0.13	0.02	0.00	0.01
Urban Mean	0.45	0.41	0.74	−0.06	−0.06	−0.05
Rural Mean	0.52	0.47	0.77	0.03	0.03	−0.07
(C) By School Size						
Large	0.062*** (0.005)	0.014*** (0.005)	0.007*** (0.002)	0.146*** (0.007)	0.107*** (0.008)	−0.074*** (0.006)
Medium	0.049*** (0.006)	0.018*** (0.005)	0.002 (0.002)	0.167*** (0.007)	0.115*** (0.008)	−0.086*** (0.006)
Small	0.060*** (0.008)	0.022*** (0.007)	0.007* (0.004)	0.129*** (0.012)	0.090*** (0.014)	−0.098*** (0.010)
P-value Difference Large-Medium	0.05	0.30	0.15	0.06	0.67	0.02
P-value Difference Large-Small	0.02	0.61	0.06	0.00	0.24	0.17
P-value Difference Medium-Small	0.69	0.35	0.66	0.03	0.14	0.05
Large Mean	0.49	0.44	0.75	−0.04	−0.03	−0.06
Medium Mean	0.53	0.48	0.77	−0.00	−0.01	−0.06
Small Mean	0.25	0.27	0.67	0.04	0.01	−0.03
N	1,895,506	1,895,436	1,850,014	983,431	991,790	1,004,899

Notes: Heteroskedasticity robust standard errors clustered by school and year are in parentheses.

* (p < .10

** p < .05

*** p < .01).

All regressions include school fixed effects and control for linear time trends in the preperiod. The regresions also control for instructional spending levels at the district, the share of students receiving free or reduced-price lunch in the school, student gender, race/ethnicity, and socioeconomic disadvantage, as well as indicators for whether the school is in a rural or urban area. High poverty schools are defined as those with at least 50% of students receiving free or reduced-price lunch. Low poverty schools are those with fewer than 50% of students receiving free or reduced-price lunch. Large schools are defined as those with at least 2000 students, medium schools have 1000 to 1999 students and small schools have fewer than 1000 students. These estimates are based on 2003–2008 and the first year of treatment is excluded. Treatment is determined by when the school first received SSCP funds. All panels include p-values reporting whether the coefficients for the different groups are significantly different from one another. Mean outcomes for each group are also reported. Estimates are reported in standard deviations for all outcomes besides overall CAHSEE pass rate.

## Footnotes

1 When estimating treatment effects on high school graduation, we focus on the 10th graders first treated by the SSCP as the first treatment cohort rather than the 12th graders. This is because the 12th graders took the CAHSEE exam and had most of the high school experiences that would influence graduation prior to when the SSCP funding arrived. Furthermore, SSCP implementation coincided with the first year in which the graduating cohort was expected to pass the exam in order to graduate, so we expect estimates for this year to include changes beyond the CAHSEE.

2 Since SAT and ACT test-taking rates are measured at the school level rather than the individual level we are unable to see whether the total number of students taking any college admissions exam changes. Instead this measure captures the number who took the SAT plus the number who took the ACT, and students who took both exams will be double counted. These results can be found in the appendix and should be interpreted with caution. For these outcomes we focus on 12th grade cohorts since we expect counselors’ advising and influence on postsecondary education to happen later in high school than their effects on high school graduation.

3 These data omit college enrollment information for about 10 percent of schools in our analytic sample. We describe differences in student demographics and outcomes for high schools with and without college enrollment data in the appendix. Table A.1 summarizes how our sample varies when omitting these schools.

4 We exclude the academic motivation index and promotion of parental involvement because the measures which comprise it were not included in the survey during our sample years. Not all questions for each index were asked in our sample year so some indices are only based on a subset of the measures in Mahecha and Hanson(2020). For students who are missing responses to component(s) of the index we take the weighted average of the nonmissing responses.

5 We refer to school years based on the fall term. For instance, the 2006–2007 academic year is referred to as 2006.

6 Due to data limitations, we do not track students across grades or exam CAHSEE exams in other grades.

7 As the tables indicate, our average treatment effect estimates are from a slightly smaller sample of 1.8 million students since we exclude the 2006–07 cohort from some estimates.

8 Thirteen schools in our school-level sample do not match to anyone in our student-level data. Students in these thirteen schools were likely removed from the sample during the data cleaning process (e.g., because they were missing student IDs or had too many duplicate observations in a given year.)

9 Results from these types of models are included in the appendix.

10 For instance, our student level CAHSEE data end in 2010 and the college enrollment data end in 2009.

11 Urbanicity refers to binary indicators for schools located in rural and urban areas as defined by the National Center for Education Statistics (NCES).Urbanicity data are missing for 2003, so we recode a given high school’s urbanicity in 2003 based on their reported urbanicity in 2004 or the first year it is recorded.

12 The survey data do not include data on socioeconomic disadvantage or receipt of free or reduced price meals.

13 This represents the average change in the number of students per counselor in the state. The causal effect of the policy change was an increase in the number of counselors per 1000 students of .578, or an equivalent reduction in the student-to-counselor ratio of more than 150 students per counselor.

14 This could reflect differences in the schools that hired counselors, since schools that used SSCP funds had a higher share of Hispanic students than schools that did not.

15 While education policy typically focuses on the student-to-counselor ratio, we report the counselor to student ratio here so that we can include schools with zero counselors.

16 However, the estimated impact on pass rates is not significant in the model without school spending and demographic controls.

17 Colleges in the University of California system are considered more selective and research intensive than those in the California State University system. UC and CSU schools are all four-year schools that predominantly offer bachelor’s degrees.

18 Gender differences for school connectedness and race/ethnicity differences for delinquency are not statistically significant.

19 Large schools are defined as those with 2000 students or more. Medium sized schools have 1000 to 1999 students and small schools have fewer than 1000 students.

20 We use this approach instead of a traditional two-way fixed effects approach with year and school fixed effects because TWFE models cannot fully account for potential violations of the pre-trends assumption and recent evidence also indicates they may be biased (e.g.,Goodman-Bacon,2021;Roth, Sant’Anna, Bilinski, & Poe,2023).

21 This type of labor market transition may have required additional licensure or certification.

## Appendix A. Additional tables and figures

See Figs. A.1–A.8 and Tables A.1–A.18.

**Table A.1:** Characteristics of High schools with and without college enrollment data.

	Missing (1)	Non-missing (2)	Difference in means (3)	P-Value (4)
Demographic Characteristics				
Share FRPL	0.479	0.396	−0.083	0.000
Female	0.414	0.490	0.076	0.000
Asian	0.026	0.077	0.051	0.000
Hispanic	0.504	0.384	−0.120	0.000
Black	0.102	0.071	−0.031	0.000
White	0.305	0.393	0.089	0.000
Rural	0.145	0.239	0.094	0.001
Urban	0.900	0.750	−0.150	0.000
High School Outcomes				
Graduation Rate	0.823	0.893	0.069	0.000
ELA	0.333	0.763	0.430	0.000
Math	0.292	0.753	0.461	0.000
Counselor Characteristics				
Counselor Ratio	3.245	2.128	−1.116	0.000
Counselors	0.690	2.846	2.155	0.000
0–5 Years of Experience	0.115	0.582	0.467	0.000
5–10 Years of Experience	0.120	0.614	0.494	0.000
10+ Years of Experience	0.454	1.649	1.195	0.000
N	241	1125		

Notes: Missing indicates schools without any available college enrollment data. P-values are reported from a ttest of the difference in means between high schools with and without college enrollment data.

## Appendix B. Sample notes

Our paper currently omits observations from LA Unified School District (LAUSD). While reviewing a prior draft of our paper, we flagged some potential issues with staffing and expenditures data for LAUSD. We observed that LA experiences an increase in counselor funding from 2009 to 2010 whereas, overall, districts see a decline in counselor funding. Further, the number of school counselors in many LA schools is implausible. No school should have more than 50 (or probably more than 15) counselors, yet high schools in LAUSD have an average of 15 counselors with a standard deviation of 24 counselors as compared to 2.63 (sd 2.57) overall. Similarly, LAUSD high schools have an average student-to-counselor of 220 (sd 250) compared to 467 (sd 370) overall. This means that 41 out of 185 high schools in LAUSD have 50 or more counselors on staff and a student-to-counselor ratio below 50; no schools outside of LAUSD have more than 50 counselors or a ratio below 50 (only 7 non-LAUSD schools have 15 or more counselors).

School finance data is reported at the district level. From 2003– 2012, LA spent $166 million on average annually (sd 21) compared to $4.3 million at other districts. LA schools spent $5.4 million on average annually (sd 9) on SSCP compared to $164,000 (sd 450k) at other districts. There do not appear to be data issues with LAUSD for other outcomes, such as high school graduation rates and test taking. Other staffing data similarly seems to be accurate for LAUSD, though there do appear to be some anomalies in instructional spending. For instance, LAUSD schools have 296 teachers on average compared to 280 for other schools. Still, instructional expenditures are way higher for LAUSD ($3.2 billion) compared to other schools ($88 million). Given the centrality of school finance data for our study, we omit LAUSD from our analytic sample.

**Table A.2:** Survey items in each index.

Characteristics
(A) Caring Staff–Student Relationships
Teacher or adult who really cares about me Teacher or adult who tells me when I do a good job Teacher or adult who notices when I’m not there Teacher or adult who always wants me to do my best Teacher or adult who listens to me when I have something to say Teacher or adult who believes that I will be a successful student

(B) School Connectedness
I feel close to people at this school. I am happy to be at this school. I feel like I am part of this school. The teachers at this school treat students fairly. I feel safe in my school.

(C) Delinquency
Been in a physical fight at school (12 months) Damaged school property on purpose at school (12 months) Carried a gun at school (12 months) Carried any other weapon at school (12 months) Been threatened or injured with a weapon at school (12 months) Seen someone carrying a gun, knife, or other weapon at school (12 months)

Notes: This table lists the survey items included in each survey index. We follow the indices and weighting recommended by the confirmatory factor analysis model described in Mahecha and Hanson (2020). If a student is missing a response for some categories of an index, we construct the index based on the non-missing items.

**Table A.3:** Summary statistics for student survey sample.

(A) Student Characteristics	
Asian	0.13
Black	0.06
Hispanic	0.40
White	0.32
Other Race	0.13
Missing Race/Ethnicity	0.01
Female	0.52
Male	0.47
Missing sex	0.01
Age	15.17
9th Grade	0.54
11th Grade	0.46
(B) School Characteristics	
School Enrollment	2205.88
Free/Reduced Lunch	0.33
Rural	0.10
Urban	0.65
HS Graduation Rate	0.92
ELA Exam	0.82
Math Exam	0.82
Students	1037473

Notes: Panel A describes the characteristics of the students who responded to the survey. Panel B describes the characteristics of the schools in which students are located (weighted by the number of students responding from each school). The exam variables refer to the average share of questions on the high school exit exam that students answered correctly.

**Table A.4:** Summary statistics for student survey sample over time.

	2003	2004	2005	2006	2007	2008	2009	2010
(A) Student Characteristics								
Asian	0.14	0.10	0.14	0.05	0.14	0.12	0.22	0.19
Black	0.05	0.06	0.06	0.04	0.06	0.06	0.08	0.16
Hispanic	0.33	0.40	0.38	0.58	0.41	0.46	0.39	0.30
White	0.37	0.34	0.33	0.26	0.30	0.28	0.23	0.05
Other Race	0.13	0.13	0.13	0.10	0.13	0.12	0.13	0.27
Missing Race/Ethnicity	0.00	0.00	0.01	0.00	0.00	0.00	0.00	0.02
Female	0.53	0.52	0.51	0.51	0.51	0.51	0.51	0.50
Male	0.46	0.47	0.48	0.48	0.48	0.48	0.48	0.49
Missing sex	0.01	0.01	0.01	0.01	0.01	0.01	0.01	0.01
Age	15.12	15.15	15.14	15.24	15.20	15.24	15.25	15.30
9th Grade	0.55	0.56	0.55	0.52	0.53	0.53	0.53	0.59
11th Grade	0.45	0.44	0.45	0.48	0.47	0.47	0.47	0.41
(B) School Characteristics								
School Enrollment	2154.14	2235.34	2252.92	1606.20	2256.58	2124.85	1908.16	1647.04
Free/Reduced Lunch	0.27	0.32	0.30	0.53	0.34	0.42	0.37	0.49
Rural	0.07	0.12	0.09	0.13	0.09	0.14	0.08	0.00
Urban	0.91	0.86	0.88	0.38	0.40	0.27	0.53	1.00
HS Graduation Rate	0.95	0.94	0.93	0.89	0.90	0.88	0.90	0.61
ELA Exam	0.82	0.79	0.83	0.77	0.83	0.82	0.83	0.75
Math Exam	0.82	0.79	0.82	0.78	0.84	0.83	0.83	0.74
Students	179963	135798	255106	6609	297389	142665	18756	1187

Notes: Panel A describes the characteristics of the students who responded to the survey over time. Panel B describes the characteristics of the schools in which students are located (weighted by the number of students responding from each school). The exam variables refer to the average share of questions on the high school exit exam that students answered correctly.

**Table A.5:** Event study results with alternatively clustered standard errors.

	Total counselors (1)	Counselor ratio (2)	High school graduation (3)	Community college enrollment (4)	CSU enrollment (5)	UC enrollment (6)	Public college enrollment (7)
(A) School-Level							
SSCP	0.908*** (0.077)	0.537*** (0.033)	0.030*** (0.002)	0.006 (0.005)	−0.003*** (0.001)	0.006*** (0.001)	0.010* (0.005)
(B) District-Level							
SSCP	0.908*** (0.112)	0.537*** (0.049)	0.030*** (0.003)	0.006 (0.010)	−0.003** (0.002)	0.006*** (0.001)	0.010 (0.011)
N	7,754,445	7,754,445	7,859,635	7,214,250	7,214,250	7,214,250	7,214,250

Notes: All regressions include school fixed effects and control for linear time trends in the preperiod, or years prior to policy implementation in 2006, and a set demographic controls: instructional spending levels at the district, the share of students receiving free or reduced-price lunch in the school, the share of students in the school who are female, the share of students in the school who are Asian, Hispanic, Black, or White, and indicators for whether the school is in a rural or urban area. Estimates in panel (A) are based on regressions where standard errors are clustered at the school level, whereas panel (B) estimates are clustered at the district level. These estimates are based on 2003–2008 and treatment is determined by when the school first received SSCP funds.

* (p < .10

** p < .05

*** p < .01).

**Table A.6:** Instrumental variables results: Effects of SSCP on academic & school climate outcomes.

	High School graduation (1)	Community college enrollment (2)	CSU enrollment (3)	UC enrollment (4)	Public college enrollment (5)	
SSCP	0.000 (0.043)	0.070 (0.050)	0.045* (0.027)	0.006 (0.010)	0.122* (0.073)	
N	3514	2841	2841	2841	2841	
	Math Scale Score (6)	ELA Scale Score (7)	CAHSEE Pass Rate (8)	Caring Staff-Student Relationships (9)	School Connectedness (10)	Delinquency (11)

SSCP	−0.369 (0.612)	−0.164 (0.445)	−0.085 (0.171)	−0.015 (.037)	0.013 (0.054)	−0.044 (0.032)
N	729,401	729,560	714,586	279,192	282,455	276,056

Notes: Results are for an instrumental variables model, where per student SSCP spending is instrumented on the number of counselors per 1000 students (i.e. our measure of student to counselor ratio). Models control for school demographic characteristics and are limited to academic years 2007–2008. Heteroskedasticity robust standard errors clustered at the school level are in parentheses.

* (p < .10

** p < .05

*** p < .01).

**Table A.7:** Intent to treat effects of SSCP on counselor, high school graduation, and college enrollment outcomes.

	Total counselors (1)	Counselor ratio (2)	High school graduation (3)	Community college enrollment (4)	CSU enrollment (5)	UC enrollment (6)	Public college enrollment (7)
(A) With Controls							
SSCP	0.929*** (0.094)	0.598*** (0.065)	0.029*** (0.003)	−0.010 (0.007)	−0.004*** (0.002)	0.010*** (0.001)	−0.004 (0.007)
(B) Without Controls							
SSCP	0.853*** (0.050)	0.532*** (0.027)	0.031*** (0.001)	0.003 (0.003)	−0.003*** (0.001)	0.008*** (0.001)	0.009*** (0.004)
N	6124	6124	5882	4462	4462	4462	4462

Notes: All regressions include school fixed effects and control for linear time trends in the preperiod, or years prior to policy implementation in 2006. Estimates in panel (A) are based on regressions which control for instructional spending levels at the district, the share of students receiving free or reduced-price lunch in the school, the share of students in the school who are female, the share of students in the school who are Asian, Hispanic, Black, or White, and indicators for whether the school is in a rural or urban area. Estimates in panel (B) exclude these controls. All estimates are based on 2003–2008 and treatment timing is the same for all schools: 2006, the first year SSCP was implemented. N reports on the number of school by year clusters. Heteroskedasticity robust standard errors clustered by school are in parentheses.

* (p < .10

** p < .05

*** p < .01).

**Table A.8:** Intent To treat estimates for high school exit exam and school climate.

	Math scale score (1)	ELA scale score (2)	CAHSEE pass rate (3)	Caring staff–student relationships (4)	School connectedness (5)	delinquency (6)
(A) With Controls						
SSCP	0.059*** (0.005)	0.009** (0.005)	0.005** (0.002)	0.139*** (0.006)	0.108*** (0.007)	−0.073*** (0.005)
(B) Without Controls						
SSCP	0.051*** (0.004)	−0.018*** (0.003)	0.001 (0.002)	0.151*** (0.003)	0.111*** (0.004)	−0.078*** (0.003)
N	1,886,346	1,886,174	1,840,425	981,292	989,711	1,002,638

Notes: Heteroskedasticity robust standard errors clustered by school and year are in parentheses.

* (p < .10

** p < .05

*** p < .01).

All regressions include school fixed effects and control for linear time trends in the preperiod. Estimates in panel (A) are based on regressions which control for instructional spending levels at the district, the share of students receiving free or reduced-price lunch in the school, student gender, student socioeconomic disadvantage, student race/ethnicity, and indicators for whether the school is in a rural or urban area. Estimates in panel (B) are from models which do not include these controls. Overall pass rate refers to passing both the math and ELA exams. Pass rates are only based on students who take the test in 10th grade. These are ITT estimates where treatment is defined to occur at the same time for everyone based on when the state policy started in 2006. Estimates are reported in standard deviations for all outcomes besides overall CAHSEE pass rate.

**Table A.9:** Effects on counselor and academic outcomes, inclusive of first year effects.

	Total counselors (1)	Counselor ratio (2)	High school graduation (3)	Community college enrollment (4)	CSU enrollment (5)	UC enrollment (6)	Public college enrollment (7)
(A) With Controls							
SSCP	0.823*** (0.060)	0.460*** (0.032)	0.031*** (0.002)	0.001 (0.004)	−0.006*** (0.001)	0.001 (0.001)	−0.004 (0.005)
(B) Without Controls							
SSCP	0.869*** (0.048)	0.509*** (0.023)	0.029*** (0.001)	0.007** (0.003)	−0.004*** (0.001)	0.004*** (0.001)	0.007** (0.003)
N	7087	7087	6880	5152	5152	5152	5152

Notes: All regressions include school fixed effects and control for linear time trends in the preperiod, or years prior to policy implementation in 2006. Estimates in panel (A) are based on regressions which control for instructional spending levels at the district, the share of students receiving free or reduced-price lunch in the school, the share of students in the school who are female, the share of students in the school who are Asian, Hispanic, Black, or White, and indicators for whether the school is in a rural or urban area. Estimates in panel (B) are from models that do not include these controls. These estimates are based on 2003–2008 and include the first year schools are treated by SSCP. Heteroskedasticity robust standard errors clustered at the school by year level are in parentheses.

* (p < .10

** p < .05

*** p < .01).

**Table A.10:** Estimates for high school exit exam and school climate, inclusive of first year effects.

	Math scale score (1)	ELA scale score (2)	CAHSEE pass rate (3)	Caring staff–student relationships (4)	School connectedness (5)	Delinquency (6)
(A) With Controls						
SSCP	0.058*** (0.004)	0.016*** (0.004)	0.005*** (0.002)	0.136*** (0.005)	0.100*** (0.005)	−0.074*** (0.004)
(B) Without Controls						
SSCP	0.051*** (0.004)	−0.016*** (0.003)	0.001 (0.002)	0.146*** (0.003)	0.106*** (0.004)	−0.078*** (0.003)
N	1,895,506	1,895,436	1,850,014	1,113,666	1,123,077	1,142,578

Notes: Heteroskedasticity robust standard errors clustered by school and year are in parentheses.

* (p < .10

** p < .05

*** p < .01).

All regressions include school fixed effects and control for linear time trends in the preperiod. Estimates in panel (B) are based on regressions which control for instructional spending levels at the district, the share of students receiving free or reduced-price lunch in the school, student gender, student socioeconomic disadvantage, student race/ethnicity, and indicators for whether the school is in a rural or urban area. Estimates in panel (B) are from models that do not include these controls. Overall pass rate refers to passing both the math and ELA exams. Pass rates are only based on students who take the test in 10th grade. These estimates are based on 2003–2008 and treatment is determined by when the school first received SSCP funds.

**Table A.11:** Estimates from Callaway and Sant’Anna approach.

	Total counselors (1)	Counselor ratio (2)	High school graduation (3)	Math scale score (4)	ELA scale score (5)	CAHSEE pass rate (6)	
ATT	0.457* (0.238)	−0.030 (0.140)	−0.006 (0.010)	0.038*** (0.012)	0.010 (0.014)	0.025*** (0.007)	
Pre-Treatment Average	.368** (.156)	.154 (.113)	−.009 (.012)	−0.010 (0.018)	0.007 (0.014)	0.013 (0.010)	
*C hi*^2^	24	38	120	170	92	52	
	Caring Staff Relationships (1)	School Connectedness (2)	Delinquency (3)	Community College Enrollment (4)	CSU Enrollment (5)	UC Enrollment (6)	Public College Enrollment (7)

ATT	0.068 (0.039)	0.030 (0.044)	0.057 (0.034)	0.083*** (0.027)	0.007 (0.004)	0.004 (0.003)	0.094*** (0.026)
Pre-Treatment Average	0.026* (0.014)	0.030 (0.024)	−0.012 (0.019)	.022 (.016)	.002 (.005)	.003 (.003)	.028 (.019)
*C hi*^2^	4	2	2	57	30	38	46

Notes: Heteroskedasticity robust standard errors clustered by school and year are in parentheses.

* (p < .10

** p < .05

*** p < .01).

These estimates represent average treatment on the treated estimates from the Callaway and Sant’Anna (2021) model using not yet treated schools or students as the comparison group. Community college, CSU, UC and Public College share refers to the percentage of seniors enrolling in community colleges, Cal State Universities, University of California institutions, and any public California college, respectively. Overall pass rate refers to passing both the math and ELA exams. Pass rates are only based on students who take the test in 10th grade. These estimates are based on 2003–2008 and include the first year of treatment. Treatment is defined based on when a school first received SSCP funds. Estimates in the bottom panel are all in standard deviations except for overall CAHSEE exam pass rates. Pre-treatment averages indicate average outcomes prior to policy implementation, and Chi2 indicates results from a joint significance test for outcomes in the preperiod.

**Table A.12:** Effects of SSCP on school outcomes, by counselor experience.

	Total counselors (1)	Counselor ratio (2)	High school graduation (3)	Community college enrollment (4)	CSU enrollment (5)	UC enrollment (6)	Public college enrollment (7)
(A) With Controls							
Increase	1.441*** (0.119)	0.873*** (0.056)	0.015*** (0.003)	−0.014* (0.008)	−0.007*** (0.002)	0.005*** (0.002)	−0.017* (0.009)
Decrease	0.873*** (0.123)	0.366*** (0.055)	0.009*** (0.003)	−0.007 (0.008)	−0.007*** (0.002)	0.006*** (0.002)	−0.008 (0.008)
No Change	0.648*** (0.110)	0.428*** (0.062)	0.007** (0.003)	−0.000 (0.008)	−0.004* (0.002)	0.008*** (0.002)	0.003 (0.009)
(B) Without Controls							
Increase	1.351*** (0.090)	0.806*** (0.040)	0.009*** (0.002)	0.002 (0.006)	−0.004*** (0.002)	0.003*** (0.001)	0.000 (0.006)
Decrease	0.786*** (0.089)	0.306*** (0.035)	0.001 (0.002)	0.012** (0.005)	−0.004*** (0.001)	0.004*** (0.001)	0.011** (0.005)
No Change	0.563*** (0.069)	0.388*** (0.042)	−0.001 (0.003)	0.012* (0.006)	−0.003** (0.002)	0.005*** (0.001)	0.013* (0.007)
N	5802	5802	5776	4267	4267	4267	4267

Notes: Coefficient estimates represent the change in outcomes of interest by whether schools that hired new counselors through SSCP spending increased, decreased, or had no change in the average years of counselor experience. All regressions include school fixed effects and controls for linear time trends in the pre-period. Estimates in panel (A) are based on regressions which control for instructional spending levels at the district, the share of students receiving free or reduced-price lunch in the school, the share of students in the school who are female, the share of students in the school who are Asian, Hispanic, Black, or White, and indicators for whether the school is in a rural or urban area. Estimates in panel (B) are from models that do not include controls. These estimates are based on 2003–2008 and treatment is determined by when the school first received SSCP funds. Heteroskedasticity robust standard errors clustered by school are in parentheses.

* (p < .10

** p < .05

*** p < .01).

**Table A.13:** Estimates for counselor & academic outcomes with continuous SSCP spending measure.

	Total counselors (1)	Counselor ratio (2)	High school graduation (3)	Community college enrollment (4)	CSU enrollment (5)	UC enrollment (6)	Public college enrollment (7)
(A) With Controls							
SSCP	0.043*** (0.005)	0.032*** (0.005)	0.000 (0.000)	0.001*** (0.000)	−0.000** (0.000)	0.000*** (0.000)	0.001*** (0.000)
(B) Without Controls							
SSCP	0.047*** (0.004)	0.038*** (0.004)	0.000** (0.000)	0.002*** (0.000)	0.000 (0.000)	0.000*** (0.000)	0.002*** (0.000)
N	5735	5735	5744	4229	4229	4229	4229

Notes: Heteroskedasticity robust standard errors clustered by school and year are in parentheses.

* (p < .10

** p < .05

*** p < .01).

All regressions include school fixed effects and control for linear time trends in the preperiod. Estimates in panel (B) are based on regressions which control for instructional spending levels at the district, the share of students receiving free or reduced-price lunch in the school, indicators for whether the school is in a rural or urban area, and indicators for whether the student is female, Asian, Hispanic, Black or White, the student’s age and grade. These estimates are based on 2003–2008 and exclude the first year schools are treated by SSCP. Treatment is determined by when the school first received SSCP funds. Estimates indicate the impact of an extra 100,000 dollars in SSCP funds.

**Table A.14:** Estimates for high school exit exam and school climate with continuous SSCP spending measure.

	Math scale score (1)	ELA scale score (2)	CAHSEE pass rate (3)	Caring staff–student relationships (4)	School connectedness (5)	Delinquency (6)
(A) With Controls						
SSCP	0.002*** (0.000)	0.001*** (0.000)	0.001*** (0.000)	0.003*** (0.001)	0.003*** (0.001)	−0.002*** (0.000)
(B) Without Controls						
SSCP	0.004*** (0.000)	−0.001*** (0.000)	0.000*** (0.000)	0.010*** (0.001)	0.008*** (0.000)	−0.005*** (0.000)
N	1,895,506	1,895,436	1,850,014	983,431	991,790	1,004,899

Notes: Heteroskedasticity robust standard errors clustered by school and year are in parentheses.

* (p < .10

** p < .05

*** p < .01).

All regressions include school fixed effects and control for linear time trends in the preperiod. Estimates in panel (A) are based on regressions which control for instructional spending levels at the district, the share of students receiving free or reduced-price lunch in the school, indicators for whether the school is in a rural or urban area, and indicators for whether the student is female, Asian, Hispanic, Black or White, the student’s age and grade. Estimates in panel (B) are from models that do not include controls. These estimates are based on 2003–2008 and exclude the first year schools are treated by SSCP. Treatment is determined by when the school first received SSCP funds. Estimates are in standard deviations and they indicate the impact of an extra 100,000 dollars in SSCP funds.

**Table A.15:** Event study results by counselor–student race/ethnicity match.

	Total counselors (1)	Counselor ratio (2)	High school graduation (3)	Community college enrollment (4)	CSU enrollment (5)	UC enrollment (6)	Public college enrollment (7)
(A) With Controls							
Race/ethnicity match: Asian	0.129 (0.217)	0.026 (0.084)	0.006 (0.004)	−0.003 (0.010)	−0.008*** (0.003)	0.005*** (0.002)	−0.005 (0.011)
Race/ethnicity match: Black	0.025 (0.241)	−0.049 (0.092)	0.014*** (0.005)	−0.002 (0.011)	−0.002 (0.003)	0.007*** (0.002)	0.003 (0.012)
Race/ethnicity match: Hispanic	1.035*** (0.164)	0.351*** (0.069)	0.018*** (0.004)	0.017 (0.010)	−0.005** (0.002)	0.010*** (0.002)	0.022** (0.011)
Race/ethnicity match: White	0.663*** (0.147)	0.278*** (0.074)	0.014*** (0.003)	−0.000 (0.010)	−0.005** (0.003)	0.005** (0.002)	−0.001 (0.010)
(B) Without Controls							
Race/ethnicity match: Asian	0.050 (0.203)	−0.052 (0.076)	−0.000 (0.003)	0.017* (0.009)	−0.005** (0.002)	0.004** (0.002)	0.016* (0.010)
Race/ethnicity match: Black	−0.036 (0.219)	−0.093 (0.080)	0.006 (0.004)	0.022** (0.010)	0.001 (0.002)	0.004** (0.002)	0.027*** (0.010)
Race/ethnicity match: Hispanic	0.891*** (0.137)	0.303*** (0.056)	0.009** (0.004)	0.037*** (0.008)	−0.003* (0.002)	0.007*** (0.001)	0.041*** (0.008)
Race/ethnicity match: White	0.660*** (0.126)	0.244*** (0.059)	0.008*** (0.002)	0.012 (0.008)	−0.003 (0.002)	0.003* (0.002)	0.011 (0.009)

Notes: Coefficient estimates represent the change in outcomes of interest for schools that hired new counselors whose race/ethnicity matches student racial demographics at that school. For instance, row 1 captures the subgroup effects of hiring an additional counselor who identified as Asian at a school with an above median percentage of students who identified as Asian. All regressions include school fixed effects and controls for linear time trends in the pre-period. Estimates in panel (A) are based on regressions which control for instructional spending levels at the district, the share of students receiving free or reduced-price lunch in the school, the share of students in the school who are female, the share of students in the school who are Asian, Hispanic, Black, or White, and indicators for whether the school is in a rural or urban area. Estimates in panel (B) are from models which exclude controls. These estimates are based on 2003–2008 and treatment is determined by when the school first received SSCP funds. Heteroskedasticity robust standard errors clustered by school are in parentheses.

* (p < .10

** p < .05

*** p < .01).

**Table A.16:** Impacts on perception of school climate.

	Adult at school cares (1)	Feel close to people at school (2)	Feel safe at school (3)	Adult tells me good job (4)	Adult notices me (5)	Adult wants what is best (6)	Adult listens to me (7)	Adult believes in me (8)
(A) With Controls								
SSCP	0.062*** (0.005)	0.065*** (0.006)	0.124*** (0.008)	0.141*** (0.005)	0.092*** (0.005)	0.171*** (0.005)	0.115*** (0.005)	0.106*** (0.005)
(B) Without Controls								
SSCP	0.064*** (0.003)	0.063*** (0.003)	0.123*** (0.005)	0.140*** (0.003)	0.092*** (0.003)	0.167*** (0.003)	0.113*** (0.003)	0.108*** (0.003)
N	964,953	980,150	979,713	965,924	965,074	961,181	964,363	962,309

Notes: Heteroskedasticity robust standard errors clustered by school and year are in parentheses.

* (p < .10

** p < .05

*** p < .01).

All regressions include school fixed effects and control for linear time trends in the preperiod, or years prior to policy implementation in 2006. Estimates in panel (A) are based on regressions which control for instructional spending levels at the district, the share of students receiving free or reduced-price lunch in the school, indicators for whether the school is in a rural or urban area, and indicators for whether the student is female, Asian, Hispanic, Black or White, the student’s age and grade. Estimates in panel (B) are from models without controls. These estimates are based on 2003–2008 and the first year of treatment is excluded. Treatment is determined by when the school first received SSCP funds.

**Table A.17:** Counselor characteristics, by school type.

	High FRPL (1)	Urban (2)	Rural (3)	All (4)
(A) Non-White Counselors				
2005	.512	.358	.298	.344
2006	.467	.377	.303	.337
2007	.477	.409	.294	.356
2008	.457	.394	.327	.353
(B) Male Counselors				
2005	.318	.289	.294	.292
2006	.286	.258	.305	.279
2007	.276	.240	.263	.259
2008	.265	.258	.259	.265
(C) Temporary Counselors				
2005	.205	.181	.274	.191
2006	.217	.187	.259	.212
2007	.252	.223	.259	.253
2008	.227	.237	.234	.230
(D) Least Experienced Counselors				
2005	.182	.169	.185	.167
2006	.220	.198	.224	.198
2007	.223	.218	.244	.232
2008	.229	.248	.247	.234

Notes: Data are limited to schools that ever used SSCP funds to hire counselors from 2006–2008. Temporary counselors refers to the proportion of counselors with temporary status; counselors are typically hired for two years before receiving tenure status. Least experienced counselors are those with 0–5 years of experience reported. High FRPL schools are those with at least 50 percent of students eligible for free or reduced price lunch.

**Table A.18:** Impacts of SSCP on total counselor spending.

	(1)	(2)	(3)
SSCP Spending	0.688*** (0.016)	0.689*** (0.017)	0.958*** (0.015)
Observations	3820	3707	3387
Limited to Program Years	Yes	Yes	Yes
Limited to Ever Treated	No	Yes	No
Limited to Treated	No	No	Yes

Notes: Coefficient estimates for SSCP Spending are taken from a regression of total counselor spending on SSCP spending and indicate how much total counselor spending increased for every dollar increase in SSCP spending (*p < .10 **p < .05 *** p < .01). All regressions include district fixed effects and are limited to program years (2006–2008) and to districts in our analytic sample.
